# Metabolomic Response of *Thalassiosira weissflogii* to Erythromycin Stress: Detoxification Systems, Steroidal Metabolites, and Energy Metabolism

**DOI:** 10.3390/plants13030354

**Published:** 2024-01-25

**Authors:** Xintong Wu, Yongqi Tong, Tong Li, Jiahua Guo, Wenhua Liu, Jiezhang Mo

**Affiliations:** 1 Guangdong Provincial Key Laboratory of Marine Disaster Prediction and Prevention, Shantou University, Shantou 515063, Chinawhliu@stu.edu.cn (W.L.); 2 Shaanxi Key Laboratory of Earth Surface System and Environmental Carrying Capacity, Northwest University, Xi’an 710127, China; jiahua_guo@nwu.edu.cn

**Keywords:** hormesis, macrolide antibiotic, algae, metabolome, steroidal compounds, toxicological mechanisms

## Abstract

Erythromycin, a macrolide antibiotic, is a prioritized pollutant that poses a high risk to environmental health. It has been detected in different environmental matrices and can cause undesired effects in aquatic organisms, particularly freshwater algae, which are primary producers. However, the impact of erythromycin on marine algae remains largely unexplored. Erythromycin has been reported to induce hormetic effects in the marine diatom *Thalassiosira weissflogii* (*T. weissflogii*). These effects are associated with the molecular pathways and biological processes of ribosome assembly, protein translation, photosynthesis, and oxidative stress. However, the alterations in the global gene expression have yet to be validated at the metabolic level. The present study used non-targeted metabolomic analysis to reveal the altered metabolic profiles of *T. weissflogii* under erythromycin stress. The results showed that the increased cell density was possibly attributed to the accumulation of steroidal compounds with potential hormonic action at the metabolic level. Additionally, slight increases in the mitochondrial membrane potential (MMP) and viable cells were observed in the treatment of 0.001 mg/L of erythromycin (an environmentally realistic level). Contrarily, the 0.75 and 2.5 mg/L erythromycin treatments (corresponding to EC_20_ and EC_50_, respectively) showed decreases in the MMP, cell density, and viable algal cells, which were associated with modified metabolic pathways involving ATP-binding cassette (ABC) transporters, the metabolism of hydrocarbons and lipids, thiamine metabolism, and the metabolism of porphyrin and chlorophyll. These findings suggest that metabolomic analysis, as a complement to the measurement of apical endpoints, could provide novel insights into the molecular mechanisms of hormesis induced by antibiotic agents in algae.

## 1. Introduction

The continuing growth of the global population drastically increases the demands for animal proteins and disease treatments, resulting in the elevated production and consumption of pharmaceuticals [1]. Consequently, the extensive presence of pharmaceuticals, especially antibiotics, in wastewater and, ultimately, in aquatic environments has become a serious concern of the scientific community [2,3]. Initially, it was believed that antibiotics only hinder the growth and proliferation of bacteria, with minimal risks to the host and other non-targeted organisms. However, accumulating evidence shows that even low levels (ng/L–µg/L) of antibiotics and/or their metabolites in the environment can have undesired effects on bacteria, phytoplankton, zooplankton, and even vertebrates [4]. Specifically, the widespread and indiscriminate use of antibiotics has led to the emergence of antibiotic resistance in bacteria. The spread of antibiotic-resistant bacteria and antibiotic resistance genes seriously threatens environmental health. Meanwhile, in some hotspots with relatively high levels of antibiotics, such as waters receiving effluents discharged from pharmaceutical factories, hospitals, and aquaculture ponds, adverse effects are widely observed in different organisms [5,6]. Decomposers and primary producers are particularly susceptible to the effects of antibiotics. Thus, the flows of substances and energy in the food web are likely to be altered under the antibiotic stress [7]. Indeed, empirical evidence has shown that exposure to antibiotics can alter the growth, physiology, and overall health of algae [5,7]. Exposure to high levels of antibiotics disrupts the signaling pathways and cellular processes necessary for algae development, ultimately inhibiting their growth. This might result in impaired photosynthesis, altered nutrition composition, reduced biomass, and decreased productivity [4,8]. It can also change the composition of algal communities by eliminating certain species of algae with high sensitivity and shifting the dominant species within the ecosystem [5]. Antibiotic stress can also indirectly affect aquatic ecosystems by disrupting interactions between algae and other organisms [2,7]. In this context, revealing the effects and mechanisms of antibiotics on algae is therefore necessary to understand their potential ecological risk [9].

Macrolides are composed of a large macrocyclic lactone ring to which one or more deoxy sugars, usually cladinose and desosamine, may be attached. The macrocyclic lactone ring gives them their antibiotic properties by allowing them to bind to the bacterial ribosome and inhibit protein synthesis [5]. Thus, macrolide antibiotics, such as erythromycin, clarithromycin, roxithromycin, and azithromycin, are extensively used to treat a variety of bacterial infections, and they have become one of the most common antibiotic categories broadly detected in surface waters and have been identified as prioritized pollutants of concern to aquatic systems with high environmental risks [5,6]. Notably, the detection of erythromycin at concentrations up to 0.076 mg/L in freshwater and up to 0.002 mg/L in coastal seawater has been documented [10,11]. Erythromycin is known to inhibit bacterial growth by suppressing protein synthesis [12]. Moreover, erythromycin exposure (µg/L–mg/L) induced prominent effects in algae, such as *R. subcapitata*, *Anabaena* sp., *Synechocystis* sp., *M. aeruginosa*, *M. flos-aquae*, *C. reinhardtii*, *I. galbana*, *C. gracilis*, *C. vulgaris*, and *S. obliquus* [13,14,15,16,17,18,19,20]. Specifically, algal growth inhibition has been linked to physiological alterations in algae exposed to erythromycin, including reduced pigment contents, photosynthesis inhibition, suppressed electron transport, inhibited lipid biosynthesis, the formation of reactive oxygen species (ROS), lipid peroxidation, and reduced cell viability [8,21,22,23,24]. These studies mainly focused on the effects of erythromycin on freshwater algae, such as green algae and cyanobacteria, but rarely on marine algal species. Nonetheless, erythromycin exposure (7.5 g/L) has been shown to reduce the chlorophyll-*a* (Chl-*a*) fluorescence and Fv/Fm and elevate the non-photochemical quenching in the seaweed *P. yezoensis* [17]. Additionally, exposure to erythromycin (mg/L) resulted in growth inhibition, a reduction in the Fv/Fm, and the elevation of ROS production in a marine diatom species (*P. tricornutum*) [18].

Diatoms, as unicellular photosynthetic eukaryotes, are found throughout the world’s marine systems [25]. They produce 20% of the world’s organic carbon through photosynthesis and contribute significantly to the global carbon cycle [26]. *Thalassiosira weissflogii* (*T. weissflogii*), also known as *Thalassiosira weissflogi*, is a species of marine diatoms belonging to the family Thalassiosiraceae. *T. weissflogii* can be found globally in various aquatic environments, including oceans, lakes, and rivers, under cold and temperate conditions [27]. As a primary producer, it plays a significant role in marine ecosystems and contributes to the global carbon cycle. One of the remarkable characteristics of *T. weissflogii* is its ability to adapt to different environmental conditions [28]. It thrives at various salinity levels and temperatures, allowing it to colonize diverse habitats. This adaptability makes it an important ecological indicator species [24]. Moreover, *T. weissflogii* serves as a vital food source for marine organisms, including zooplankton, which provide nutrition for higher trophic levels in the marine food web [29]. Additionally, it plays a crucial role in producing organic matter through photosynthesis, contributing to carbon fixation and regulating the levels of atmospheric CO_2_ [28,29]. Indeed, *T. weissflogii* has been used as a model marine diatom to evaluate the ecotoxicity of various contaminants (e.g., metals, nanoparticles, herbicides, and pesticides) for environmental risk assessment [29,30,31,32,33]. However, barely any ecotoxicological information on antibiotics in this species is available. Most recently, a hormetic effect (algal growth was promoted in low-dose treatments but inhibited at high-dose exposures) of erythromycin on *T. weissflogii* was reported, in which the molecular signaling pathways and biological processes related to xenobiotic metabolism, ribosome assembly, protein translation, photosynthesis, and oxidative stress were modified [34]; however, these transcriptional and physiological changes have yet to be validated at the metabolic level. Nonetheless, it has been proposed that the mechanisms underlying growth promotion induced by low-level exposure to chemicals/pollutants are attributed to an increase in ROS production, pigment contents, protein synthesis, and possibly the accumulation of phytohormones [35,36]. Whether the accumulation of phytohormones or phytohormone-like substances is involved in the erythromycin-induced growth promotion has yet to be clarified.

Metabolomics, relying on the techniques of nuclear magnetic resonance or mass spectrometry, can be employed to profile all the metabolites of a given biological system in a high-throughput manner [37]. This omics technology can probe into organism–environment interactions at the molecular level and decipher the metabolic pathways altered by abiotic and/or biotic stresses [38].

Based on recently published transcriptomic data [34], this study hypothesizes that erythromycin treatments modify the metabolic pathways involved in ribosome assembly, amino acid biosynthesis and/or metabolism, and photosynthesis. We investigated the effects of erythromycin stress on *T. weissflogii* by analyzing its metabolomic (non-targeted metabolome) and biochemical responses, such as its mitochondrial membrane potential (MMP) and cell membrane integrity. To validate the transcriptomic alterations in *T. weissflogii* under erythromycin stress, the metabolomic data generated in the present study were conjointly analyzed with the published transcriptomic data [34]. This study aimed to gain a better understanding of erythromycin ecotoxicity in marine algae, identify potential cellular targets, and develop biomarkers for erythromycin toxicity in marine diatoms.

## 2. Results

### 2.1. Erythromycin Exposure Modified the Growth of Marine Microalga

**Figure 1:**
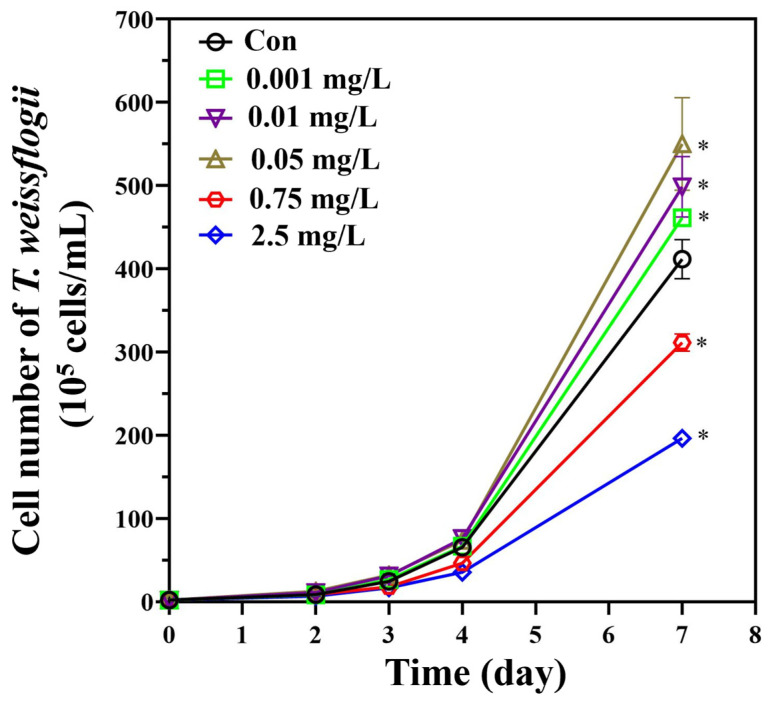
Hormetic effects in *T. weissflogii* induced by erythromycin treatments. Data are presented as mean ± SD (*n* = 3), and an asterisk (*) signifies statistical significance (*p* < 0.05).

The growth of *T. weissflogii* under erythromycin stress is shown in Figure 1. The results presented here were in accordance with the published data [34], where a hormetic effect was observed in *T. weissflogii* under erythromycin stress. Specifically, the algal cells of the 0.001, 0.75, and 2.5 mg/L erythromycin groups were 112.1% ± 2.4%, 75.6% ± 2.5%, and 47.7% ± 1.8% of those of the control (Figure 1).

### 2.2. Erythromycin Exposure Modified MMP and Caused Algal Cell Death

**Figure 2:**
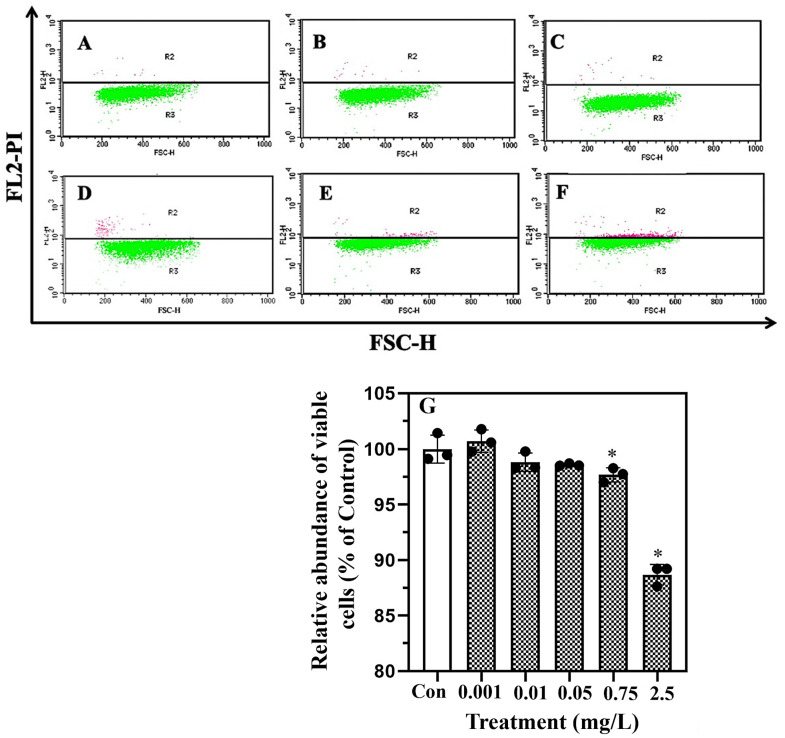
Erythromycin treatments induced algal cell death. Stained algal cells with higher and lower propidium iodide (PI) fluorescence intensities in sections R2 and R3 signify the alive and dead cells, respectively (**A**–**F**) Control, 0.001, 0.01, 0.05, 0.75, and 2.5 mg/L erythromycin treatment groups. In the bar chart (**G**), data are presented as mean ± SD (*n* = 3), and an asterisk (*) signifies statistical significance (*p* < 0.05).

Viable algal cells were distinguished from dead cells by identifying changes in the cell membrane integrity in the PI staining assay. In Figure 2A–F, the *X*-axis represents the forward scatter (FSC) that detects scatter along the path of the laser and indicates the cell size, while the *Y*-axis shows the fluorescence intensity of the PI detected via the flow cytometry analysis. Stained algal cells with higher and lower PI fluorescence intensities in sections R2 and R3 signify the alive and dead cells, respectively. The proportion of viable cells dropped significantly (*p* < 0.05) by 2.5% ± 0.6% and 11.3% ± 0.9% in the 0.75 and 2.5 mg/L erythromycin treatments, respectively, in comparison with the control (Figure 2G).

**Figure 3:**
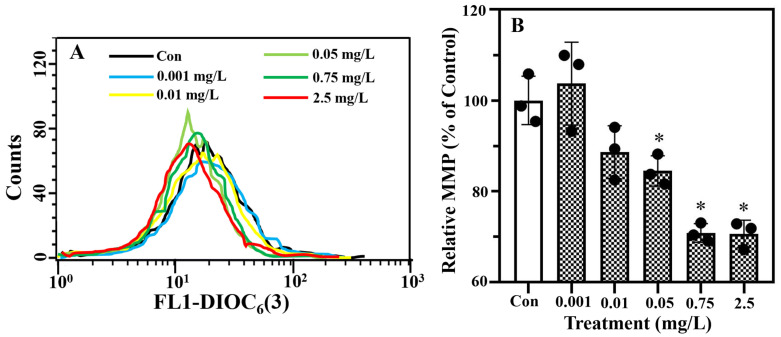
Evaluation of mitochondrial membrane potential (MMP) of *T. weissflogii* exposed to erythromycin with DIOC_6_(3) staining using a flow cytometer. The DIOC_6_(3) fluorescence in the algal populations (**A**) and the relative MMP bar chart (**B**) are presented. Data are presented as mean ± SD (*n* = 3), and an asterisk (*) signifies statistical significance (*p* < 0.05).

Additionally, alterations in the MMP of *T. weissflogii* following erythromycin exposure were examined via DIOC_6_(3) staining using flow cytometry. In Figure 3A, the *X*-axis and *Y*-axis represent the fluorescence intensity of the PI and the cell number, respectively. A higher fluorescence intensity indicates a higher MMP of the algal cells. The relative MMP decreased significantly (*p* < 0.05) in the *T. weissflogii* treated with erythromycin at concentrations above 0.05 mg/L (Figure 3B). Specifically, it was reduced by 29.2% ± 2.0% and 29.4% ± 3.0% in the 0.75 and 2.5 mg/L erythromycin treatments.

### 2.3. Erythromycin Exposure Altered Metabolomic Profiles

For the metabolomic profiles, 466 and 302 metabolites with clear identities were recorded using the positive and negative modes, respectively. The correlations of the QC samples in both the positive mode (0.994–0.995) and negative mode (0.995–0.997) are shown in Figure S1. Unsupervised PCA was then employed to evaluate the variations in the metabolomic profiles (Figure S2). In both modes, replicates of the QC samples clustered, indicating that high-quality metabolomic data were generated in this study. In addition, it was observed that replicates of the control and the 0.001 mg/L erythromycin treatment were closely clustered, while the other two erythromycin treatment groups were separated from them. This finding is consistent with the growth promotion experienced by *T. weissflogii* when exposed to 0.001 mg/L of erythromycin, whereas growth suppression was observed in the 0.75 and 2.5 mg/L erythromycin groups (Figure 1).

According to the multivariate statistical analysis (OPLS-DA), the R2Y and Q2 were determined to be 0.997–1 (0.999–1) and 0.784–0.959 (0.572–0.943) for the negative (positive) mode, respectively. Notably, clear separations were evident in the OPLS-DA score plots of the metabolites detected in both the negative and positive modes between the treatment groups (Figure S3). As illustrated in Table S1, totals of 8 (3 upregulated, 5 downregulated), 26 (21 upregulated, 5 downregulated), and 36 (23 upregulated, 13 downregulated) DAMs were identified in the negative mode in the 0.001, 0.75, and 2.5 mg/L erythromycin treatments, respectively, when a threshold VIP > 1 and *p* < 0.05 in the Student’s *t*-test were applied. In the positive mode, there were 20 (16 upregulated, 4 downregulated), 64 (60 upregulated, 4 downregulated), and 124 (102 upregulated, 22 downregulated) DAMs identified in the 0.001, 0.75, and 2.5 mg/L erythromycin groups, respectively (Table S2). These DAMs, identified either in the negative mode or positive mode, were collectively subjected to the KEGG pathway analysis. Specifically, 1, 8, and 12 KEGG pathways were significantly (*p* < 0.05) enriched in *T. weissflogii* treated with 0.001, 0.75, and 2.5 mg/L of erythromycin, respectively (Table 1).

### 2.4. Conjoint Analysis of Metabolomic and Transcriptomic Data

The metabolomic data and transcriptomic data were conjointly analyzed. In total, 1, 7, and 19 DAMs were paired with at least one transcript in *T. weissflogii* treated with 0.001, 0.75, and 2.5 mg/L of erythromycin compared to the control, respectively (Table S3). These pairs of DAMs and transcripts were mapped to multiple KEGG pathways, which include histidine metabolism, ether lipid metabolism, glycerophospholipid metabolism, thiamine metabolism, ABC transporters, cysteine and methionine metabolism, drug metabolism—cytochrome P450, etc.

## 3. Discussion

The present study delineated the erythromycin-induced effects in a marine microalga with novel metabolomic data. Partially in agreement with the hypothesis, erythromycin (mg/L) exerted its toxicity by modifying the metabolic pathways involved in the detoxification, amino acid metabolism, and photosynthesis systems. Notably, amino acid metabolism emerged as a key target of erythromycin ecotoxicity in *T. weissflogii*, while the energy metabolism in both the mitochondria and chloroplasts was perturbed by high-dose erythromycin stress. As novel metabolomic data, the accumulation of steroidal compounds with potential hormonal action was highlighted in *T. weissflogii* with growth enhancement induced by erythromycin (μg/L).

### 3.1. Detoxification Systems

The induction of hormesis in algae by erythromycin has been attributed to the differential actions of xenobiotic metabolism at different erythromycin exposure levels [14,15,34]. Apart from the treatment dose, the toxicity of erythromycin is also determined by the detoxification capacity of algae and, thus, by the cellular retention time and action of erythromycin. Xenobiotic metabolism in plants like algae typically comprises redox reactions, the introduction of hydrophilic groups, and elimination [39,40]. ABC transporters, as exporters, are responsible for the exportation of metabolized xenobiotics into the vacuole, cell walls, and/or external environment [41]. ABC transporters can also act as intracellular transporters for various substrates, such as vitamins, amino acids, lipids, secondary metabolites, carbohydrates, etc., crossing organelles by consuming ATP [42].

In this study, metabolic pathways responsible for the metabolism and elimination of xenobiotics, such as drug metabolism—cytochrome P450 and ABC transporters, were enriched in the 0.75 mg/L erythromycin treatment. In comparison, ABC transporters were significantly altered in the 2.5 mg/L erythromycin treatment. Specifically, in the 0.75 mg/L erythromycin treatment group, the accumulations of thiamine and choline were associated with the upregulation of the ABC transporter genes *abca3* and *abcb1*. By contrast, the accumulated contents of thiamine, choline, glutamate, glycerol, and phosphate in the 2.5 mg/L erythromycin treatment were linked to the upregulation of genes encoding the ABCA subfamily (*abca3*) and ABCB subfamily (*abcb1* and *atm*). These results suggest that perturbations in substance transportation and energy metabolism (discussed in Section 4.2) occurred in *T. weissflogii* under the stress of high levels of erythromycin, albeit the upregulation of ABC transporters might be beneficial to the detoxification of erythromycin in *T. weissflogii*. Substance transportation through ABC transporters, both intracellularly and extracellularly, is energy-demanding (i.e., consumption of ATP), and less energy is likely available for the growth (e.g., protein synthesis, DNA replication, silicon deposition, etc.) and division of *T. weissflogii*, which is supported by the decreased MMP, reduced growth, and increased cell death in the 0.75 and 2.5 mg/L erythromycin treatments. The data presented here are partially in line with the metabolic alterations in *R. subcapitata* under erythromycin stress, in which the contents of metabolites, including carbohydrates, amino acids, and nucleoside-related compounds, were drastically modified and associated with the actions of ABC transporters [15].

Interestingly, metabolic pathways associated with xenobiotic metabolism and detoxification were not affected in the group treated with 0.001 mg/L of erythromycin. This implies that the crucial factor in determining the effects of erythromycin is the treatment dose, rather than the detoxification capacity of the algae. This is because erythromycin is relatively resistant to degradation in algal toxicity tests, where from 56.2% (±11.1%) to 76.4% (±8.8%) of the initial erythromycin remained in the media after a 7-day exposure [34].

### 3.2. Energy Metabolism

Carbohydrates, lipids, and amino acids are essential cellular components and energy carriers [43]. After glycolysis in cytoplasm, most of the energy (i.e., ATP) is produced within mitochondria through the TCA cycle and oxidative phosphorylation using various energy substances generated from a complexed network involved in the metabolisms of carbohydrates, lipids, and amino acids [44,45]. Pathways related to the metabolism of carbohydrates (e.g., the citrate cycle, glyoxylate and dicarboxylate metabolism, and carbon fixation pathways in prokaryotes) were consistently modified in the 0.75 and 2.5 mg/L erythromycin treatments. In these metabolic pathways, a reduction in isocitrate was associated with the downregulation of *aco* encoding aconitate hydratase (Figure S4). This suggests that the normal flow of the TCA cycle was inhibited. Furthermore, treatment with 2.5 mg/L of erythromycin caused a disturbance in oxidative phosphorylation. An increase in the phosphate content was linked to the exclusive downregulation of genes that encode V-type ATPase (e.g., *atpev1a*, *atpev1b*, *atpev1c*, *atpev1d*, *atpev1e*, *atpev1f*, *atpev1h*, *atpev0a*, *atpev0c*, *atpev0d*). According to the omics data, the MMP decreased distinctly in the *T. weissflogii* treated with 0.75 and 2.5 mg/L of erythromycin. However, further in-depth investigations on the functional impacts of erythromycin on *T. weissflogii*, such as respiration, are still warranted. Taken together, these results suggest that the energy metabolism was likely impaired both in the chloroplasts and mitochondria of the *T. weissflogii* following exposure to high levels of erythromycin, ultimately resulting in an elevation in ROS formation [34,46], a reduction in the cell membrane integrity, and growth inhibition.

In the present study, pathways related to lipid metabolism (e.g., ether lipid metabolism and glycerophospholipid metabolism) were enriched in the 0.75 and 2.5 mg/L erythromycin treatments. Additionally, the metabolic pathway of glycerolipid metabolism was altered in the 2.5 mg/L erythromycin treatment. Specifically, the accumulations of glycerophosphocholine, phosphocholine, and choline in *T. weissflogii* were associated with the consistent upregulation of *lypla2* (encodes lysophospholipase II), *gde1* (encodes glycerophosphodiester phosphodiesterase), and *cki1* (encodes choline kinase). As choline accumulation has been linked to improved osmoprotection and resistance to abiotic stresses in plants [47,48], these metabolic alterations were likely a positive feedback of *T. weissflogii* to combat the erythromycin toxicity. These findings in *T. weissflogii* partially accord with the modified metabolic pathways, including carbohydrate metabolism and lipid metabolism (but not purine metabolism or pyridine metabolism) in *R. subcapitata* [15], suggesting that they might adopt different strategies to adjust their energy metabolism and apportion to tackle the erythromycin toxicity.

Notably, steroid hormone biosynthesis was the one signaling pathway altered in the 0.001 mg/L erythromycin treatment group, in which significant accumulations of 5.alpha.-pregnane-3.alpha.,20.alpha.-diol (26.9 folds) and 5alpha-androstan-17beta-ol-3-one (27.3 folds) were recorded (Table 1). Animal sex hormones have long been found to be present in plants, but little is known about their biosynthesis and conversion in plants [49]. Previous studies have shown that these two steroidal metabolites could exert hormonic activity in animals [50,51,52], albeit their functions in plants, including algae, remain unknown. Nevertheless, fish steroidal hormones, 17β-estradiol, and 17,20,β-dihydroxy-4-pregnen-3-one were demonstrated to promote the growth of *Scenedesmus quadricauda*, associated with the accumulation of Chl-*a*, carotenoids, and lipids [53]. Therefore, we hypothesize that these metabolites might serve as phytosterol-like compounds [54,55,56,57], stimulating algal growth under low-dose erythromycin stress; however, subsequent studies are required to test this hypothesis and reveal other key events, such as lipid metabolism and the resulting lipid profiles using lipidomic analysis, in erythromycin-induced growth promotion. Additionally, the accumulation of phytohormones and the resulting promoted growth in plants might be due to the increased ROS production induced by low-dose stressors [58]. Whether and how the slight increase in the ROS production in *T. weissflogii* under low-dose erythromycin stress [34] contributed to the accumulation of the steroidal compounds identified in the present study warrant further investigations. Intriguingly, steroid hormone biosynthesis was also modified in *T. weissflogii* treated with 0.75 mg/L of erythromycin, but the contents of 5.alpha.-pregnane 3.alpha.,20.alpha.-diol (1.4 folds), 5alpha-androstan-17beta-ol-3-one (1.6 folds), and aldosterone (1.8 folds) were only slightly elevated. This implies a threshold for these steroidal metabolites to exert their stimulative actions on *T. weissflogii*, and only at concentrations above the threshold can they lead to profound growth promotion. Further in-depth studies on the biosynthesis and conversion of steroidal compounds in plants will improve our understanding of the mechanisms underlying chemical-induced hormesis in plants [59].

It has been proposed that alterations in the ribosome assembly, translation, and soluble protein contents are responsible for the observed dose-dependent erythromycin-induced effects in *T. weissflogii* [34]. In this study, there were one (histidine metabolism) and two (glycine, serine, and threonine metabolism and cysteine and methionine metabolism) pathways involved in amino acid metabolism that were modified in the 0.001 and 0.75 mg/L erythromycin treatments, respectively. In contrast, when treated with 2.5 mg/L of erythromycin, a total of nine amino acid metabolism pathways were modified in the treatment: histidine metabolism; arginine and proline metabolism; alanine, aspartate, and glutamate metabolism; glycine, serine, and threonine metabolism; tyrosine metabolism; cysteine and methionine metabolism; arginine biosynthesis; D-glutamine and D-glutamate metabolism; and aminoacyl-tRNA biosynthesis. Our data suggest that amino acid metabolism was a primary target disturbed in *T. weissflogii* under erythromycin stress. While the contents of most amino acids were not affected in any of the three erythromycin treatment groups, both L-glutamate and D-glutamine, intriguingly, were accumulated in the 2.5 mg/L erythromycin treatment, correlated with the upregulation of *glud1_2* (encoding glutamate dehydrogenase, which can convert 2-Oxoglutarate into L-glutamate). One possible explanation is that the inhibition of porphyrin and chlorophyll metabolism led to the accumulation of L-glutamate, an initial substrate for chlorophyll production, through a series of enzymatic catalysis actions (discussed in Section 4.3). Alternatively, it might serve as positive feedback for producing proline and combating erythromycin toxicity. Indeed, abiotic stresses have been shown to induce ROS formation and inhibit the activities of glutamate dehydrogenases, leading to the accumulation of glutamate, the acceleration of proline synthesis, and improvement in the resistance to abiotic stresses in high plants [60,61].

Taken together, the accumulations of amino acids (e.g., L-glutamate, D-glutamine), lipids (e.g., glycerol, stearidonic acid, linolenic acid, PC (16:0/16:0)), and phosphoric acid in high-dose erythromycin treatments suggested that the *T. weissflogii* was facing an energy crisis in which lipids and amino acids were catabolized to supply energy (i.e., ATP). This, in turn, could have profound impacts on the physiology of *T. weissflogii*, as most of the energy is used for combating the erythromycin toxicity and for survival instead of for growth (e.g., protein synthesis, DNA replication, silicon deposition, etc.) and proliferation.

### 3.3. Thiamine Metabolism and Metabolism of Porphyrin and Chlorophyll

Thiamine, as a cofactor of energy metabolism-related enzymes, is required for carbohydrate metabolism, NADPH, ATP, and nucleic acid pentoses during cell growth and development [62,63,64]. Moreover, thiamine accumulation has been shown to increase the resistance to abiotic stresses in high plants [63]. In this study, molecular pathways related to thiamine biosynthesis and metabolism (i.e., thiamine metabolism and the sulfur-relay system) were enriched in the 0.75 and 2.5 mg/L erythromycin treatments. Notably, thiamine accumulation was associated with the downregulation of *iscS* (encodes cysteine desulfurase). During thiamine biosynthesis, sulfur transfer is achieved via several enzymatic reactions catalyzed by cysteine desulfurase, tRNA uracil 4-sulfurtransferase, and sulfur carrier protein ThiS adenylyltransferase. The elevated thiamine content here in the *T. weissflogii* likely served as an attempt to combat or alleviate the erythromycin-induced toxicity in both the 0.75 and 2.5 mg/L erythromycin treatments.

In photosynthetic organisms like algae, chlorophylls are biosynthesized via multiple enzymatic reactions and are key components of light-harvesting complexes [65,66]. In this study, the reduced content of pheophytin-*a* was linked to a handful of genes that encode enzymes of porphyrin and chlorophyll metabolism. Specifically, *ears* (encodes glutamyl-tRNA synthetase), *hemA* (encodes glutamyl-tRNA reductase), *hemL* (encodes glutamate-1-semialdehyde 2,1-aminomutase), *hemB* (encodes porphobilinogen synthase), *hemC* (encodes hydroxymethylbilane synthase), *hemD* (encodes uroporphyrinogen-III synthase), *hemE* (encodes uroporphyrinogen decarboxylase), *hemF* (encodes coproporphyrinogen III oxidase), *hemY* (encodes protoporphyrinogen/coproporphyrinogen III oxidase), *chlH* (encodes magnesium chelatase subunit H), *dvr* (encodes divinyl chlorophyllide a 8-vinyl-reductase), and *por* (encodes protochlorophyllide reductase) were exclusively downregulated (Figure S5), implying that the biosynthesis of porphyrin and chlorophyll were completely shut down due primarily to photosynthesis inhibition and the excessive production of ROS in the 2.5 mg/L erythromycin group [34,67]. Moreover, the light-harvesting dysfunction produced inadequate ATP, as supported by the accumulation of phosphoric acid. It thus was unable to support physiological processes, including carbon fixation and other cellular metabolic reactions [68], amplified as the suppression of the protein synthesis [34], growth inhibition, and cell death of *T. weissflogii*. Notably, the downregulation of *cox10* (encodes heme o synthase), *cox15* (encodes heme a synthase), and *hccs* (encodes cytochrome c heme-lyase) implies the dysfunction of the metabolism of heme A and cytochrome c, both of which are critical components of the electron respiratory transport chain within the mitochondria [69]. This was supported by the reduction in the algal MMP in the 2.5 mg/L erythromycin treatment at the physiological level. The dysfunction of mitochondria and impairments of energy metabolism contributed to the growth suppression and cell death of *T. weissflogii* in response to high levels of erythromycin. Further studies on the respiration of *T. weissflogii* under erythromycin stress are required to probe into the linkages between the functional impacts and metabolomic alterations.

### 3.4. Environmental Implications

Antibiotic pollution is now a pressing environmental issue, as it can exert undesired effects on non-target organisms [2]. Accumulating experimental evidence has shown that exposure to antibiotics induces hormesis in various algal species (e.g., Cyanophyta, Chlorophyta, and Bacillariophyta), in which low doses of antibiotics can enhance performances, such as the growth, pigment content, protein content, etc., while high doses inhibit them [34,35,54,55]. Diatoms play a vital role in maintaining the ecological balance in the oceans and the overall stability of the earth’s ecosystem through their contributions to oxygen production and the chemical balance in the ocean (e.g., silicon cycling, carbon sequestration), and by serving as the foundation of the marine food chain [25]. Changes in diatoms induced by antibiotic stress at the population, cellular, and metabolomic levels might have ecological consequences. In the present study, the exposure of *T. weissflogii* to erythromycin at 0.001 mg/L induced a growth promotion, associated with the accumulation of steroidal metabolites, in addition to an elevation in the soluble proteins and Chl-*a* [34]. This implied that erythromycin at environmentally realistic levels (μg/L) could potentially increase the biomass and abundance of algae like *T. weissflogii*, and their nutrition values (e.g., rich in soluble proteins and Chl-*a*) would likely improve. In this context, organisms in the higher trophic levels of food chains (e.g., zooplankton, shellfish, etc.) might benefit from it, as they might have a better chance of preying on algae with relatively high nutrition values. However, a potential health risk is that these algae might contain certain levels of antibiotics and their residues, causing dietary exposures to these grazers [6]. Notably, it has been reported that erythromycin induced hormesis in toxin-producing cyanobacteria (e.g., *M. aeruginosa* and *M. flos-aquae*), which seriously threaten environmental health [13,19,23]. Exposure to high levels of erythromycin (mg/L) is unlikely to happen on an ecological scale, except in certain hotspots, like aquaculture areas. In certain extreme scenarios, *T. weissflogii* exposed to erythromycin at a certain milligram per liter might show a reduction in its growth, biomass, and contents of soluble proteins and Chl-*a* [34]. At the same time, metabolites, including free amino acids, choline, thiamine, etc., are accumulated. This is very likely to cause adverse effects on the zooplankton that feed on *T. weissflogii*, as their preferred foods are in lower abundance and have degraded nutrition values. All these alterations in *T. weissflogii* induced by erythromycin might ultimately affect the structure and function of the food chains in marine ecosystems [7]. Other ecological services of *T. weissflogii*, such as oxygen production, silicon cycling, and carbon sequestration, are likely to be affected under erythromycin stress. However, subsequent studies performed in ecological environments are warranted to verify these ecological consequences.

## 4. Materials and Methods

### 4.1. Algal Cultivation and Toxicity Testing

This work was performed using the marine diatom *T. weissflogii* (CCMP 1587), which originated from the Provasoli-Guillard National Center for the Culture of Marine Phytoplankton (CCMP, United States). The marine microalga was seeded in the f/2 medium (artificial seawater with a 30‰ salinity) and cultured under optimal conditions (22 ± 1 °C, 80 μmol photons m^−2^ s^−1^, and a cycle of 12 h:12 h = light:dark). *T. weissflogii* (1 × 10^5^ cells/mL) in the logarithmic growth phase was seeded in an erythromycin exposure medium within a conical flask. Based on the dose–response data of erythromycin to *T. weissflogii* that were published recently [34], the metabolomic and biochemical responses of *T. weissflogii* to erythromycin at concentrations of 0.001 mg/L (environmentally relevant level), 0.75 mg/L (EC_20_), and 2.5 mg/L (EC_50_) were assessed in the present study. Accordingly, a freshly prepared erythromycin stock solution (10 mg/L) was diluted with the culture media to achieve the nominated erythromycin exposure concentrations at the initiation of the exposure experiment (day 0), and the *T. weissflogii* was exposed to erythromycin at 0.001, 0.75, and 2.5 mg/L for 7 days. During the entire exposure, the algal media was neither renewed nor supplemented with erythromycin. To determine the growth performance of *T. weissflogii* with or without erythromycin treatment, algal cells were counted using a hemacytometer under a microscope on days 2, 3, 4, and 7 during the experiments. On day 7, the samples collected from the erythromycin treatments and the control were used for subsequent biochemical measurements or metabolomic analysis.

### 4.2. Determination of Biochemical Parameters

Cell death was determined by the intactness of the cell membranes using propidium iodide (PI) staining [70]. Additionally, the MMP of *T. weissflogii* was determined using 3,3′-dihexyloxacarbocyanine iodide (DiOC_6_(3)) following a standard protocol [71]. In brief, *T. weissflogii* was centrifugally collected (3000× *g*, 10 min) and treated with 10 µM propidium iodide (20 min, in the dark) or 2.5 μM DiOC_6_(3) (30 min, in the dark). The stained cells were ultimately analyzed using flow cytometry, with excitations of 488 nm/emission 620 nm and 484 nm/emission 500 nm applied to analyze the propidium iodide and DiOC_6_(3), respectively.

### 4.3. Metabolomic Profiling

The metabolomic analysis was executed on the collected algal samples as described previously [72]. Briefly, a solution (400 μL) composed of methanol, acetonitrile, and water with a volume ratio of 4:4:2 was used to extract metabolites from *T. weissflogii*. After thorough mixing through a vortex, the extraction was performed at −20 °C for 60 min. The resulting extracts were centrifugated (14,000× *g*, 20 min, 4 °C) to collect supernatants for vacuum drying and redissolution in acetonitrile–water solution (100 μL). Following centrifugation (14,000× *g*, 15 min, 4 °C), the separation of samples was performed in a chromatographic column (1.7 μm, 2.1 mm × 100 mm; Agilent Technologies, United States). The gradient program of separation is illustrated in Table S4. Finally, the separated substances were detected with a TripleTOF 6600 mass spectrometer (AB SCIEX, United States) linked to an electron spray ionization (ESI) under positive and negative modes (detailed in the supplementary data). Equal volumes (10 μL) of samples were obtained from both control and erythromycin treatments and mixed to make quality control (QC) samples. Both testing samples and QC samples were randomly analyzed at 4 °C. As illustrated in Figure S6, the response intensity of the total ion current signal overlapped well, suggesting good instrument stability and the high validity of the results generated. The resulting raw data were converted into mzXML format using Proteo Wizard, while the peak alignment, retention time correction, and peak area extraction were executed using the XCMS program. Subsequently, standards database was used to identify the metabolites detected in the samples based on the primary and secondary information of the mass spectra generated. Multidimensional statistical analyses, such as principal component analysis (PCA) and orthogonal partial least-squares discriminant analysis (OPLS-DA), were conducted using SIMCA-P 14.1. To ensure the reliability of the applied model, a permutation test was conducted 200 times (Figure S7). Differentially accumulated metabolites (DAMs) were identified using the threshold, a variable influence on projection (VIP) > 1, and *p* < 0.05 in the Student’s *t*-test. All DAMs identified in both analysis modes were ultimately subjected to the Kyoto Encyclopedia of Genes and Genomes (KEGG) pathway analysis using MetaboAnalyst (version 5.0).

### 4.4. Conjoint Analysis of Metabolome and Transcriptome

The metabolomic data were conjointly analyzed with the transcriptomic data [34]. DAMs and transcripts with a *p* < 0.05 were extracted from these two data sets and mapped onto the KEGG pathway database to generate the regulatory metabolomic pathways.

### 4.5. Statistical Analysis

Data were analyzed using Graph Pad Prism 9.2. A normality test was executed on the data on algal growth and cell death. A one-way analysis of variance (ANOVA) followed by Tukey’s post hoc test was applied, and a statistical difference (*) was deemed when a *p* value < 0.05 was achieved.

## 5. Conclusions

The alterations in the metabolomic profiles of *T. weissflogii* caused by erythromycin exposure were dose-dependent. Algal growth promotion in the 0.001 mg/L erythromycin treatment group was linked to accumulated steroidal metabolites with potential hormonic action. Contrarily, the algal growth suppression observed in the 0.75 and 2.5 mg/L erythromycin treatments might have been the result of the dysregulation of metabolic pathways, including ABC transporters, energy metabolism, thiamine metabolism, and the metabolism of porphyrin and chlorophyll. Exposure to high levels of erythromycin induced the dysfunction of chloroplasts and mitochondria. This study showed that metabolomic analysis could provide novel insights into the molecular mechanism(s) of antibiotics like erythromycin, and it can serve as a complement to the measurement of apical endpoints. Although exposure to an environmentally relevant level of erythromycin (0.001 mg/L) exerted a growth promotion in *T. weissflogii* under controlled conditions in this laboratory study, whether this effect can be induced in algae in ecological environments (e.g., considering environmental and biological factors) and the possible ecological consequences have yet to be verified. The hormetic effects observed in *T. weissflogii* and *R. subcapitata* [15] suggested that low doses of erythromycin promoted algal growth and production, which enhances the potential of algae as biodiesel and biomass feedstocks used in the industry [54].

## Figures and Tables

**Table 1:** Metabolic pathways (*p* < 0.05) in *T. weissflogii* altered by erythromycin treatments. DAMs: differentially accumulated metabolites.

Pathways	Map ID	Map Name	*p* Value	Down-DAMs	Up-DAMs
**Control vs. 0.001 mg/L erythromycin treatment**					
Lipid metabolism	ko00140	Steroid hormone biosynthesis	0.040	-	5.alpha.-pregnane-3.alpha.,20.alpha.-diol; 5alpha-androstan-17beta-ol-3-one
**Control vs. 0.75 mg/L erythromycin treatment**					
Lipid metabolism	ko00590	Arachidonic acid metabolism	0.001	-	5s-hydroxy-6e,8z,11z,14z-eicosatetraenoic acid; Leukotriene f4; Prostaglandin i2; Prostaglandin a2; PC (16:0/16:0)
Lipid metabolism	ko00564	Glycerophospholipid metabolism	0.001	-	PC (16:0/16:0); Phosphocholine; Choline; Glycerophosphocholine
Amino acid metabolism	ko00330	Arginine and proline metabolism	0.004	-	Caldine; Guanidoacetic acid; DL-Glutamic acid
Membrane transport	ko02010	ABC transporters	0.005	-	Thiamine; N-acetyl-d-glucosamine; Choline; DL-Glutamic acid
Lipid metabolism	ko00592	alpha-Linolenic acid metabolism	0.005	-	Linolenic acid; Stearidonic acid; PC (16:0/16:0)
Amino acid metabolism	ko00260	Glycine, serine, and threonine metabolism	0.008	-	Ectoine; Choline; Guanidoacetic acid
Biosynthesis of other secondary metabolites	ko00332	Carbapenem biosynthesis	0.028	-	DL-Glutamic acid
Lipid metabolism	ko00140	Steroid hormone biosynthesis	0.046	-	5.alpha.-pregnane 3.alpha.,20.alpha.-diol; 5alpha-androstan-17beta-ol-3-one; Aldosterone
**Control vs. 2.5 mg/L erythromycin treatment**					
Membrane transport	ko02010	ABC transporters	1.89 × 10^−5^	-	Phosphoric acid; Thiamine; L-glutamate; D-glutamine; Sucrose; Glycerol; N-acetyl-d-glucosamine; Deoxyadenosine; Choline
Amino acid metabolism	ko00330	Arginine and proline metabolism	0.001	Caldine; Guanidoacetic acid	L-glutamate; G-guanidinobutyrate; gamma-aminobutyric acid
Amino acid metabolism	ko00250	Alanine, aspartate, and glutamate metabolism	0.001	-	L-glutamate; D-glutamine; gamma-aminobutyric acid
Translation	ko00970	Aminoacyl-tRNA biosynthesis	0.002	-	L-glutamate; D-glutamine
Amino acid metabolism	ko00220	Arginine biosynthesis	0.007	-	L-glutamate; D-glutamine
Lipid metabolism	ko00590	Arachidonic acid metabolism	0.009	-	5s-hydroxy-6e,8z,11z,14z-eicosatetraenoic acid; Prostaglandin i2; PC (16:0/16:0); 15-deoxy-delta-12,14-pgj2; Lipoxin a4
Lipid metabolism	ko00564	Glycerophospholipid metabolism	0.012	-	Phosphocholine; PC (16:0/16:0); Glycerophosphocholine; Choline
Metabolism of other amino acids	ko00471	D-Glutamine and D-glutamate metabolism	0.020	-	L-glutamate; D-glutamine
Global and overview maps	ko01230	Biosynthesis of amino acids	0.022	Isocitric acid	D-glutamine; L-glutamate
Cell growth and death	ko04111	Cell cycle—yeast	0.034	-	Phosphoric acid
Lipid metabolism	ko00592	alpha-Linolenic acid metabolism	0.039	-	Stearidonic acid; Linolenic acid; PC (16:0/16:0)
Energy metabolism	ko00910	Nitrogen metabolism	0.041	-	D-glutamine; L-glutamate

## Data Availability

The data generated in this work are available to those interested upon request.

## Supplementary Materials

The following supporting information can be downloaded at https://www.mdpi.com/article/10.3390/plants13030354/s1, Figure S1: The correlation of metabolites for quality control samples in both modes; Figure S2: Principal component analysis of metabolomic profiles in *T. weissflogii* under erythromycin stress; Figure S3: Metabolic profiles evaluated by orthogonal projections to latent structures discriminant analysis; Figure S4: Citrate cycle modified in *T. weissflogii* under erythromycin stress; Figure S5: Metabolism of porphyrin and chlorophyll modified in *T. weissflogii* exposed to 2.5 mg/L erythromycin; Figure S6: The overlaid chromatograms of three quality control samples; Figure S7: Permutation tests for samples in different erythromycin treatments compared with the control; Table S1: Identification of differential metabolites in the negative mode; Table S2: Identification of differential metabolites in the positive mode; Table S3: Integrated analyses of differentially accumulated metabolites and genes in *T. weissflogii* treated with erythromycin; Table S4: The gradient program of ultra-high performance liquid chromatography used for sample separation.
