# Supplementary material for: Metabolomic Response of Thalassiosira weissflogii to Erythromycin Stress: Detoxification Systems, Steroidal Metabolites, and Energy Metabolism
Source: Plants (Basel). 2024 Jan 25;13(3):354. doi: 10.3390/plants13030354 (PMC10856835; doi:10.3390/plants13030354)
Supplement: Supplementary file 1 [file plants-13-00354-s001.zip › plants-2772986-supplementary.pdf]

Supplementary Materials

Metabolomic Response of *Thalassiosira weissflogii* to Erythromycin Stress: Detoxification Systems, Steroidal Metabolites, and Energy Metabolism

Xintong Wu [1,†], Yongqi Tong [1,†], Tong Li [1], Jiahua Guo [2], Wenhua Liu [1] and Jiezhang Mo [1,*]

[1] Guangdong Provincial Key Laboratory of Marine Disaster Prediction and Prevention, Shantou University, Shantou 515063, China; whliu@stu.edu.cn (W.L.)
[2] Shaanxi Key Laboratory of Earth Surface System and Environmental Carrying Capacity, Northwest University, Xi'an 710127, China; jiahua_guo@nwu.edu.cn
* Correspondence: jzhmo@stu.edu.cn; Tel.: +86-183-2039-0437
† These authors contributed equally to this study.

LC-MS/MS analysis and data processing

After the extraction of metabolites, subsequent analyses were performed using an UHPLC (Agilent Technologies, USA) coupled to a quadrupole time-of-flight (AB Sciex TripleTOF 6600, USA). The program used for the separation was detailed in **Table S4**. The ESI source conditions were applied for the detection: Ion Source Gas1 (Gas1): 60, Ion Source Gas2 (Gas2): 60, curtain gas (CUR): 30, source temperature: 600℃, IonSpray Voltage Floating (ISVF) ± 5500 V.

In MS only acquisition, the m/z range and accumulation time for time-of-flight mass spectrometry (TOF MS) scan was set at 60-1000 Da and 0.20 s/spectra, respectively. In auto MS/MS acquisition, the instrument was set to acquire over the m/z range 25-1000 Da, and the accumulation time for product ion scan was set at 0.05 s/spectra, according to the information dependent acquisition (IDA) with high sensitivity mode selected. The parameters set for analysis were as follow: the collision energy (CE) was fixed at 35 V with ± 15 eV; declustering potential (DP), 60 V (+) and −60 V (−); exclude isotopes within 4 Da, candidate ions to monitor per cycle: 10.

The raw data of mass spectrum (wiff.scan files) were initially converted to MzXML files using ProteoWizard MSConvert and analyzed using the XCMS software (Sciex, USA). For peak picking, parameters including centWave m/z = 10 ppm, peakwidth = c (10, 60), prefilter = c (10, 100) were set, while

parameters (i.e., bw = 5, mzwid = 0.025, and minfrac = 0.5) were applied in peak grouping. Subsequently, CAMERA (Collection of Algorithms of MEtabolite pRofile Annotation) was employed to annotate the isotopes and adducts. In the extracted ion features, only the variables that had more than 50% of the nonzero measurement values and were presented at least in one treatment group (the control and/or erythromycin treatments) were retained. Compound identification of metabolites was performed by comparing of accuracy m/z value (< 10 ppm), and MS/MS spectra with standards database.

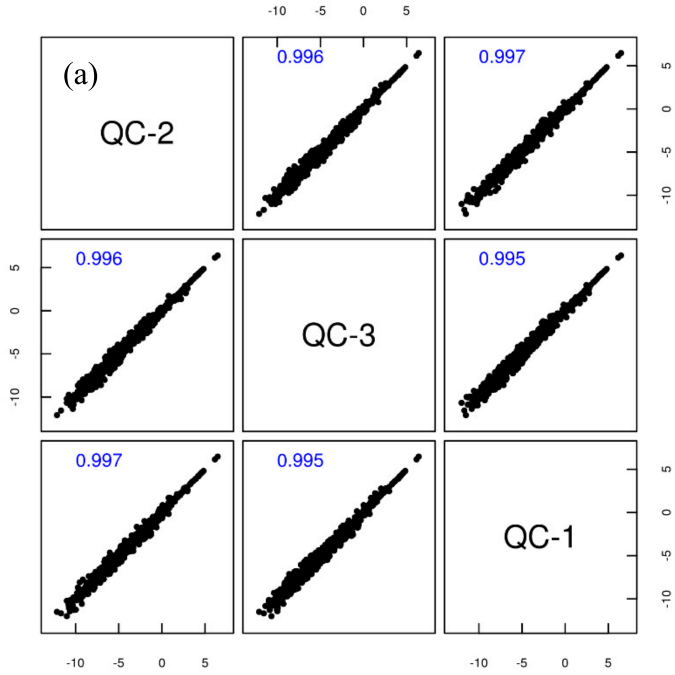

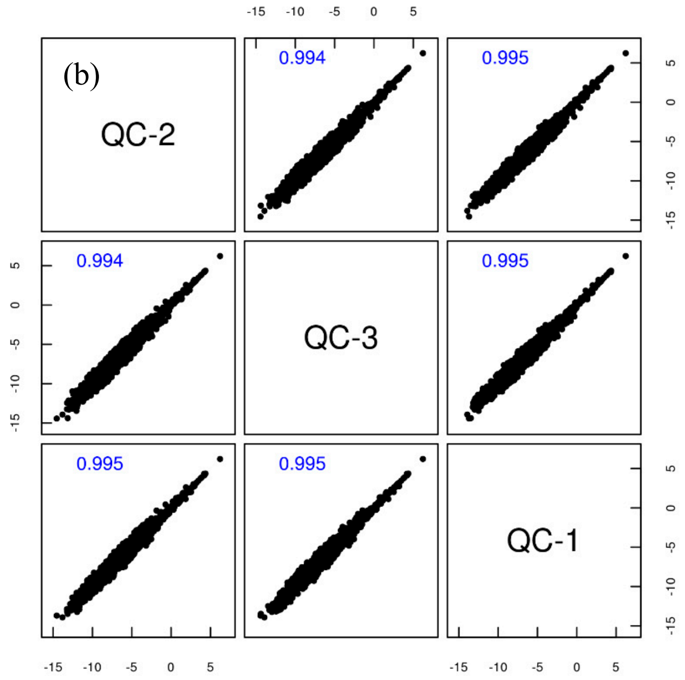

Figure S1. The correlation of metabolites for quality control (QC) samples in negative mode (a) and positive mode (b).

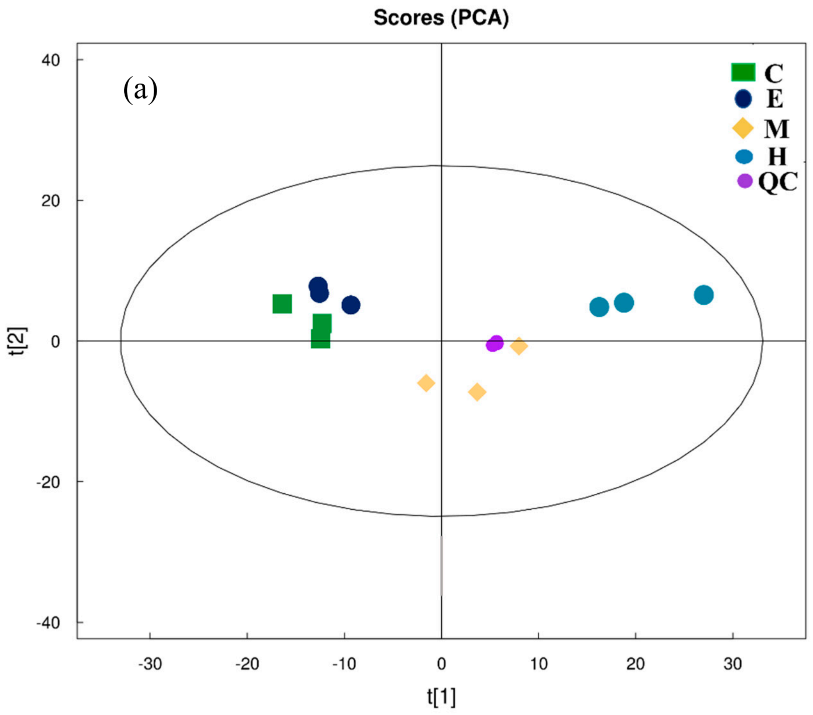

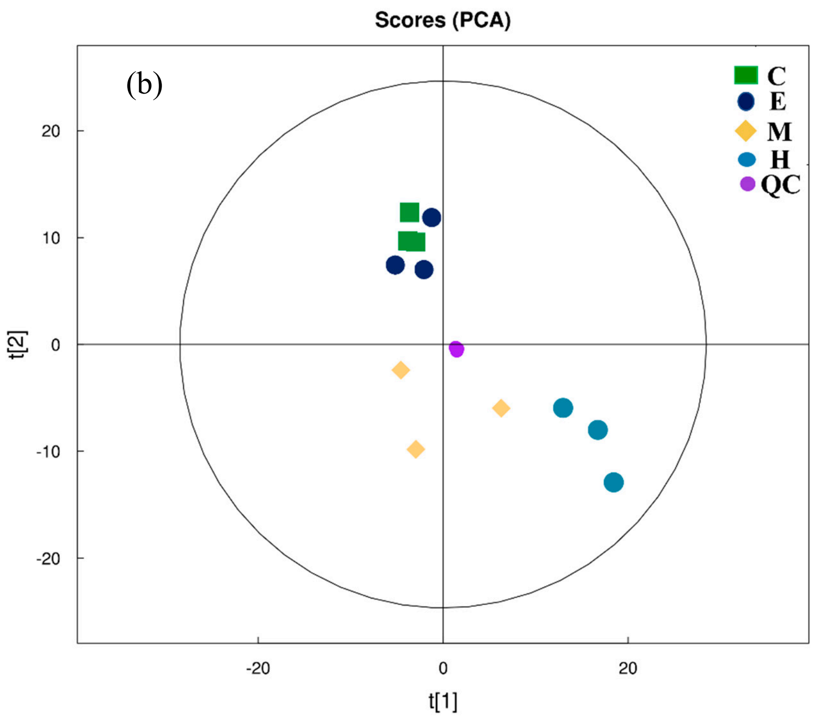

Figure S2. Principal component analysis (PCA) of metabolomic profiles in *T. weissflogii* altered by different erythromycin treatments. Metabolites were detected in negative ion mode (a) and positive ion mode (b). C (green squares), E (red dots), M (yellow squares), H (blue dots) and QC (purple dots) represents the control, 0.001, 0.75, 2.5 mg/L erythromycin treatment and quality control (QC), respectively.

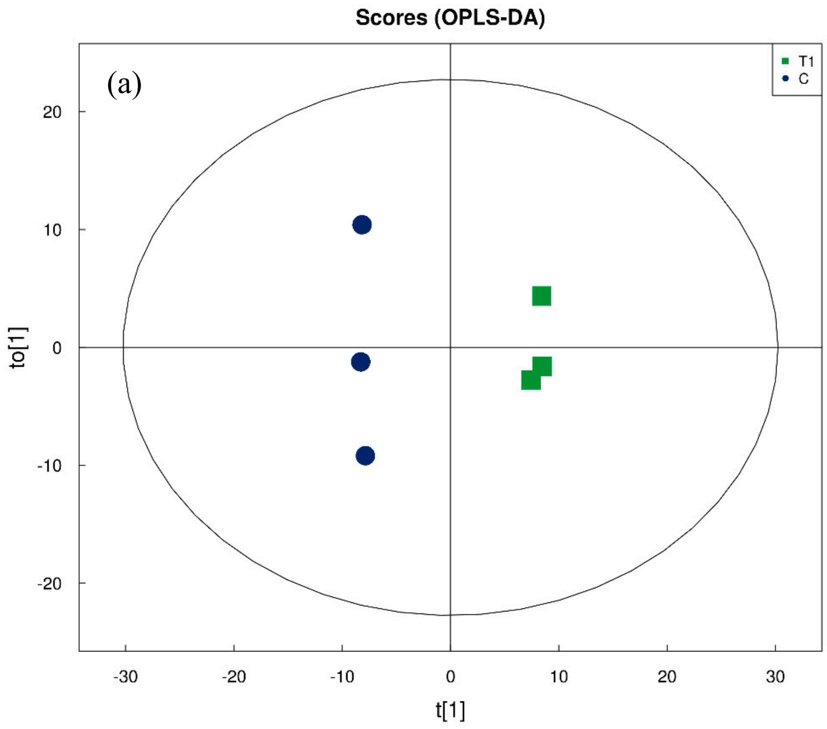

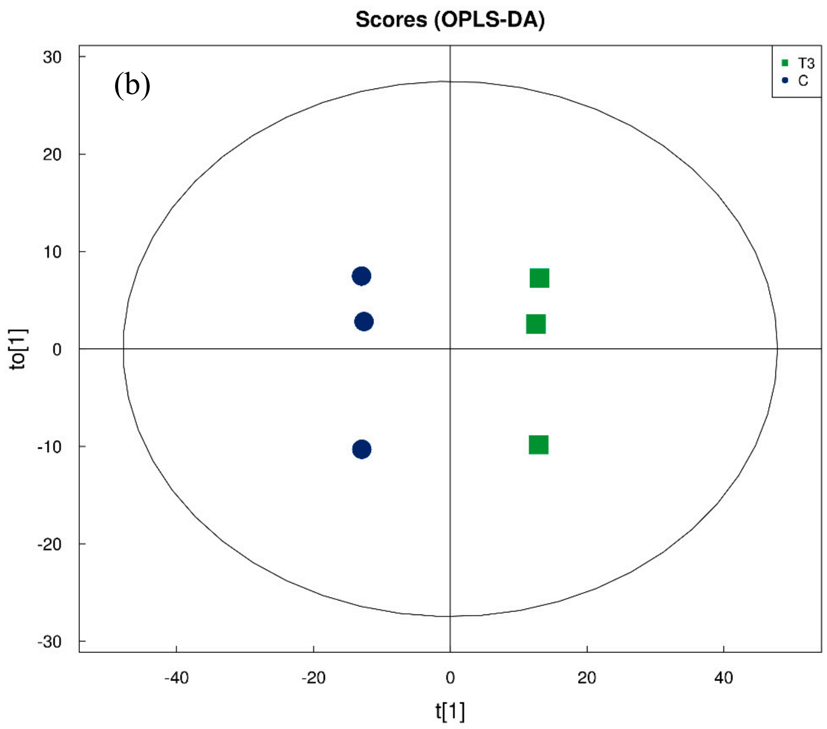
Scores (OPLS-DA)
(b)
T3
C
30
20
10
0
-10
-20
-30
to[1]
-40
-20
0
20
40
t[1]

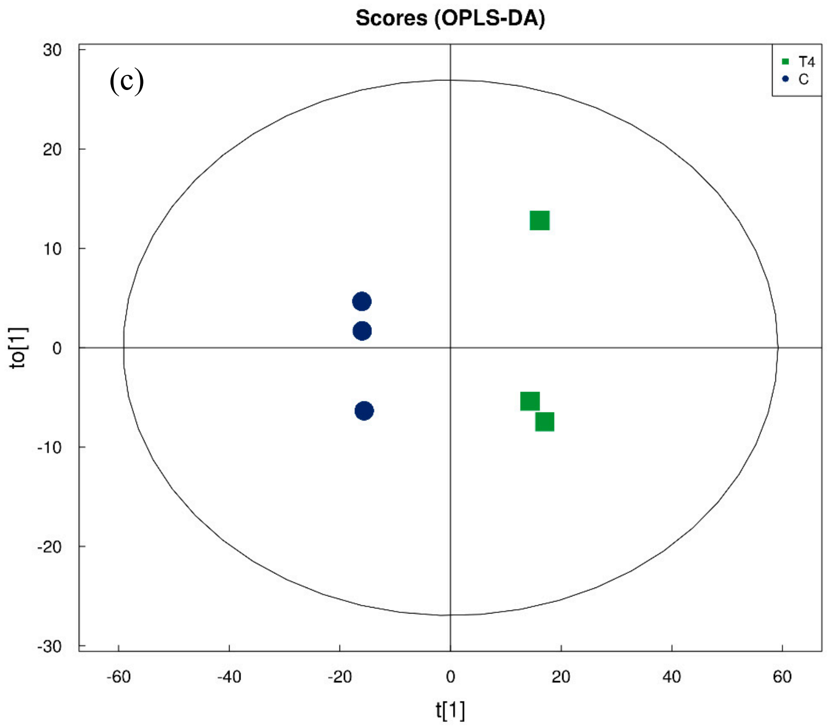
Scores (OPLS-DA)
(c)
T4
C
30
20
10
0
-10
-20
-30
to[1]
-60
-40
-20
0
20
40
60
t[1]

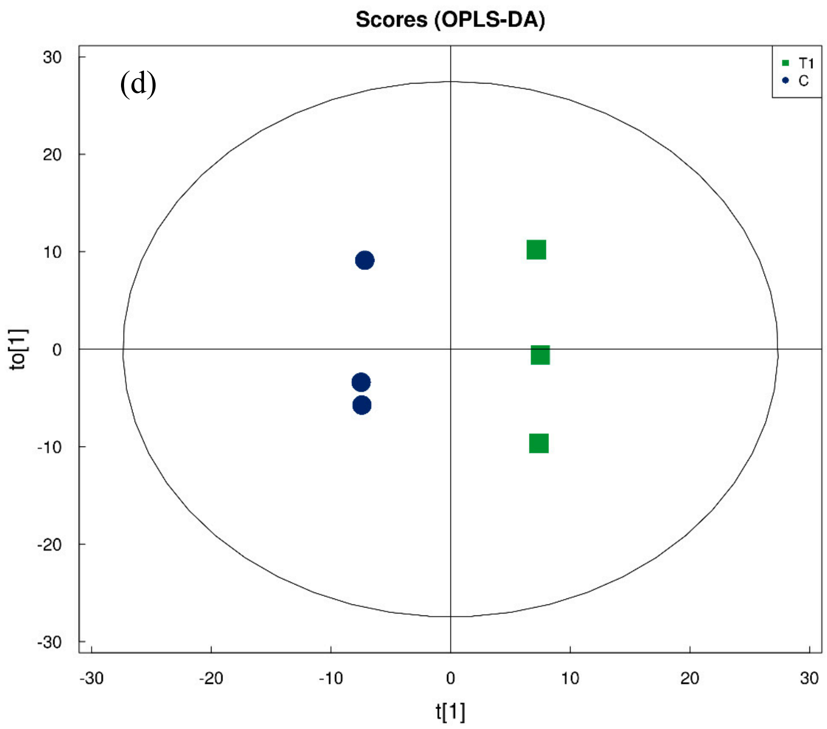
Scores (OPLS-DA)
(d)
T1
C
to[1]
t[1]
30
20
10
0
-10
-20
-30
-30
-20
-10
0
10
20
30

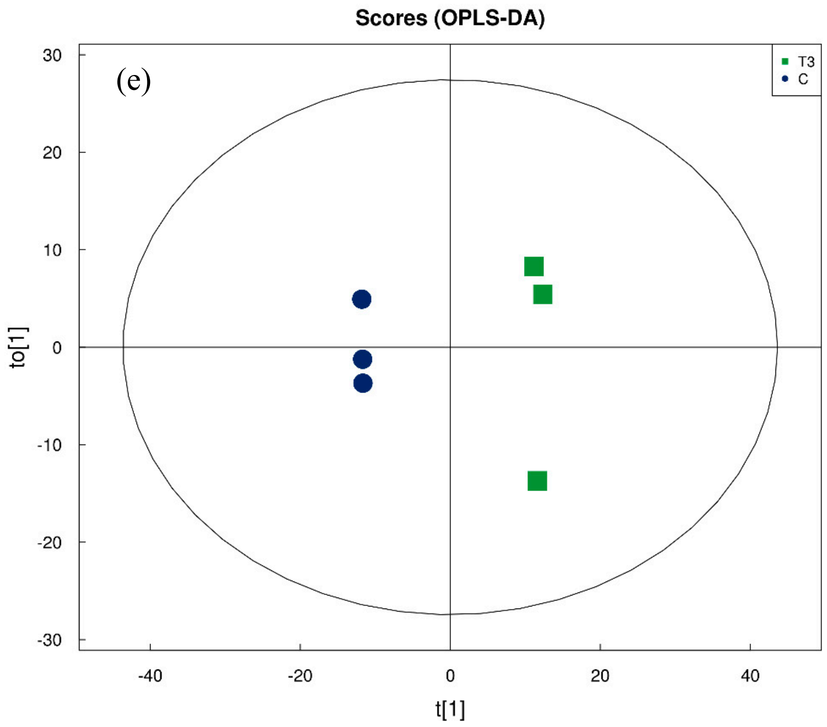
Scores (OPLS-DA)
(e)
T3
C
to[1]
t[1]
30
20
10
0
-10
-20
-30
-40
-20
0
20
40

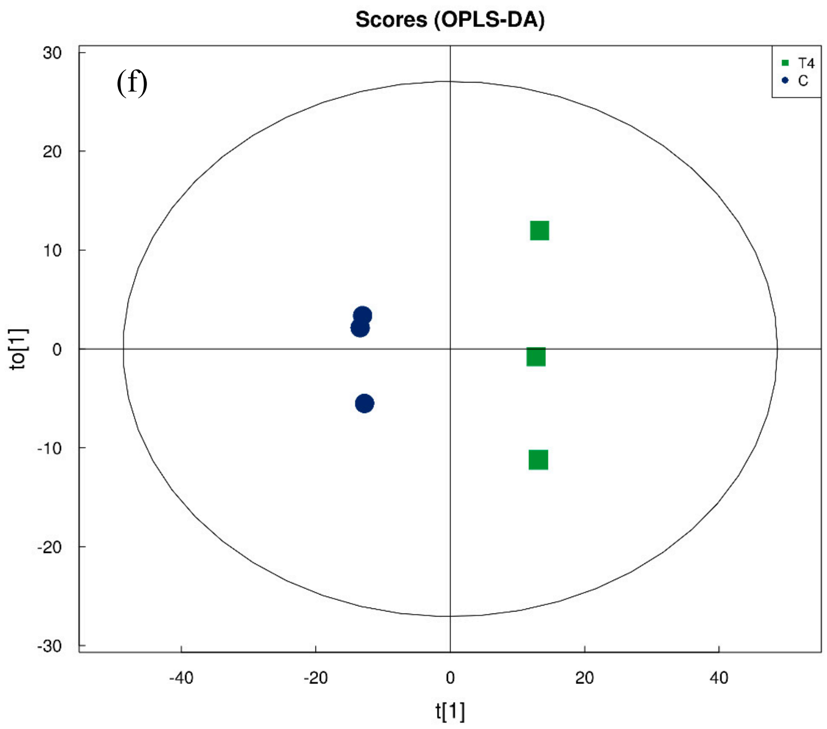

Figure S3. Metabolic profiles of *T. weissflogii* following erythromycin treatments evaluated by orthogonal projections to latent structures discriminant analysis (OPLS-DA). OPLS-DA score plots of metabolites detected in the negative (a, b and c: 0.001, 0.75, and 2.5 mg/L erythromycin treatment compared to the control, respectively) and positive (d, e and f: 0.001, 0.75, and 2.5 mg/L erythromycin treatment compared to the control, respectively) ion mode.

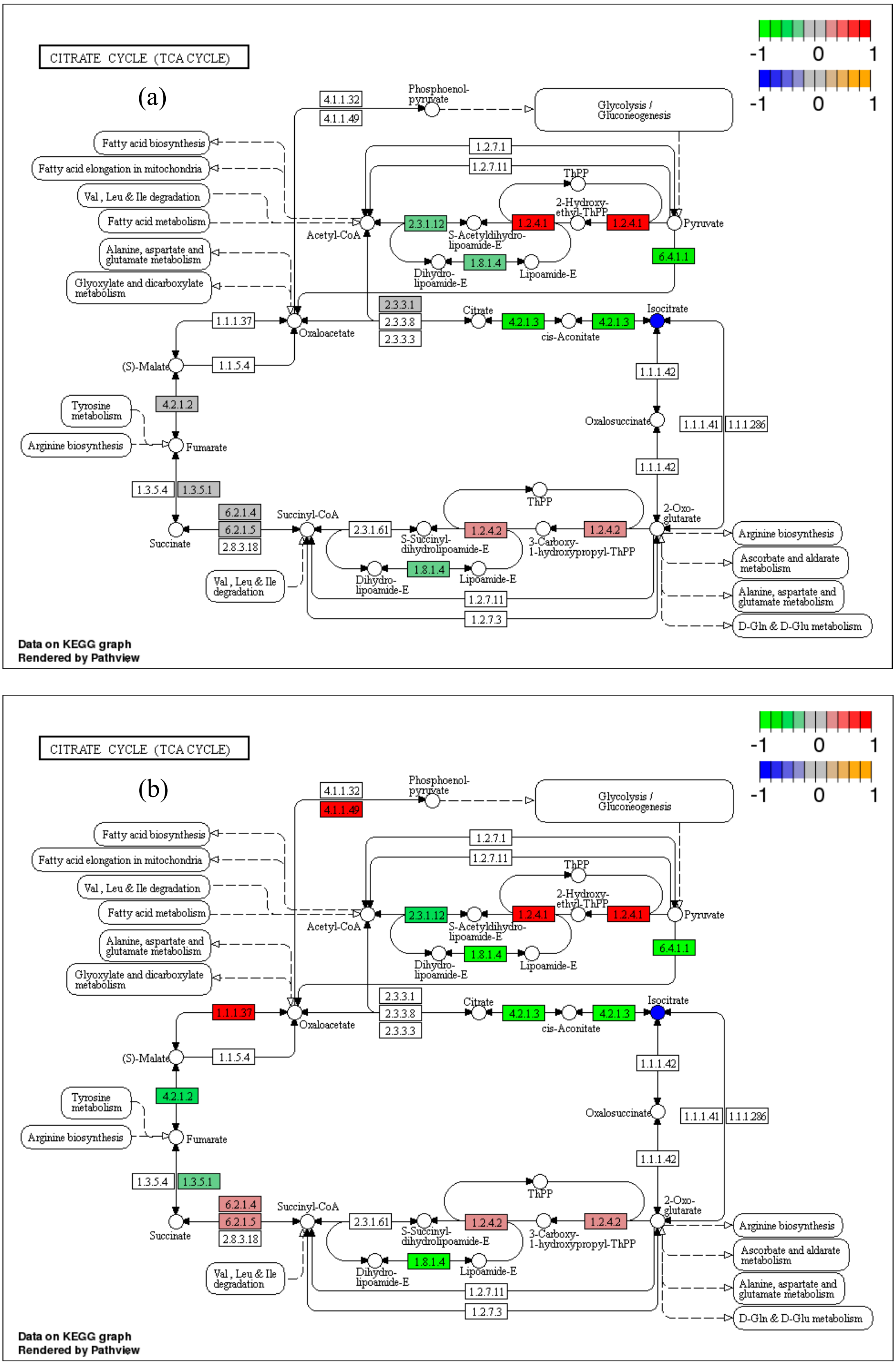

Figure S4. Citrate cycle (TCA cycle) modified in *T. weissflogii* under erythromycin stress. Differentially accumulated metabolites and differentially expressed transcripts are highlighted in the 0.75 mg/L (a) and 2.5 mg/L (b) erythromycin treatments.

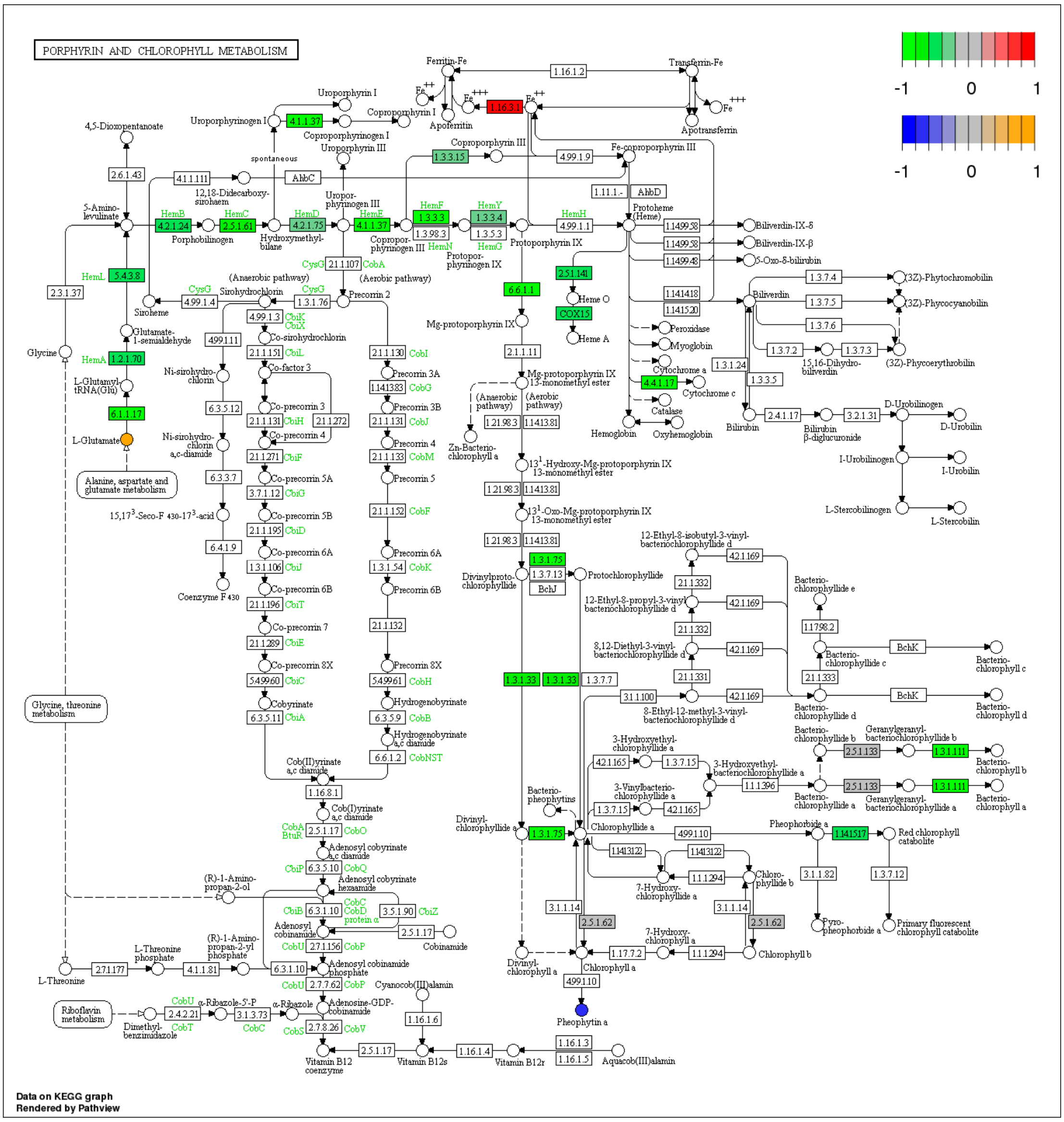

Figure S5. Metabolism of porphyrin and chlorophyll modified in *T. weissflogii* exposed to 2.5 mg/L erythromycin. Differentially accumulated metabolites and differentially expressed transcripts are shown in the metabolic network.

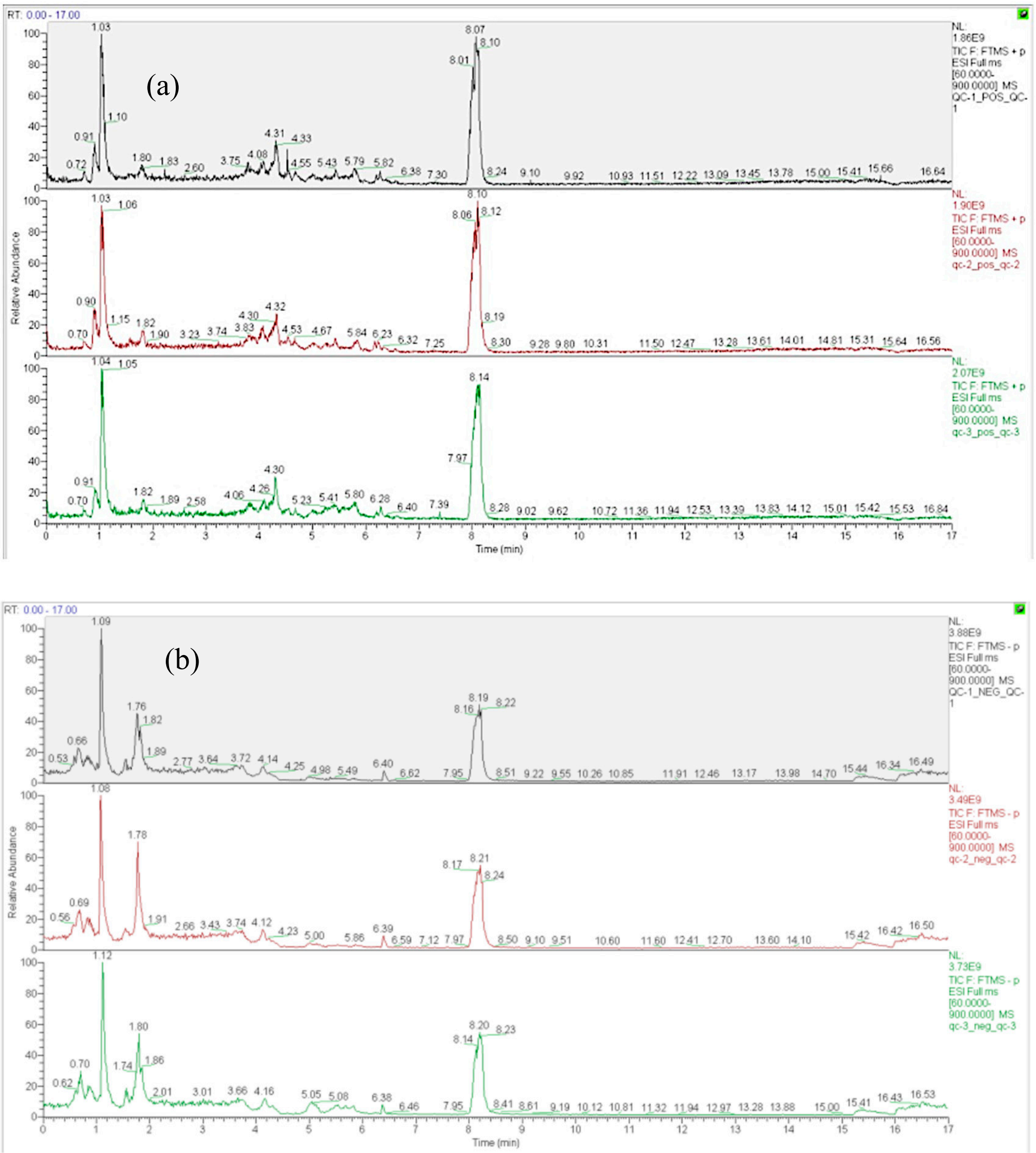

Figure S6. The retention time and total ion current signal intensity reproducibility was determined using overlaid chromatograms of three quality control (QC) samples. The total ion chromatogram overlaid mass spectrum of positive ion mode (a) and negative ion mode (b).

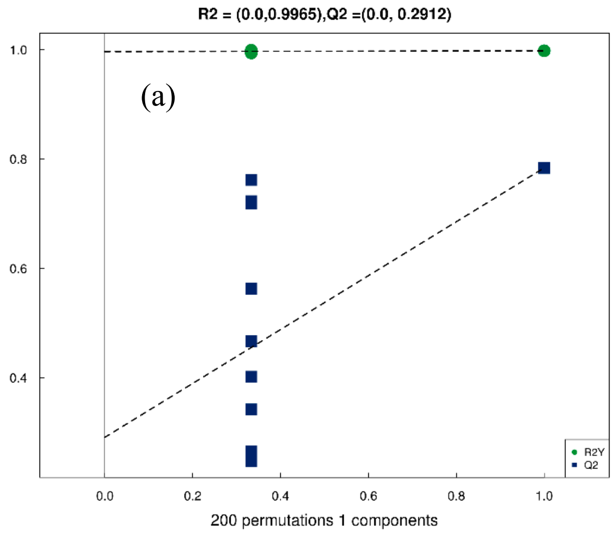
R2 = (0.0,0.9965),Q2 =(0.0, 0.2912)
(a)
1.0
0.8
0.6
0.4
0.0
0.2
0.4
0.6
0.8
1.0
R2Y
Q2
200 permutations 1 components

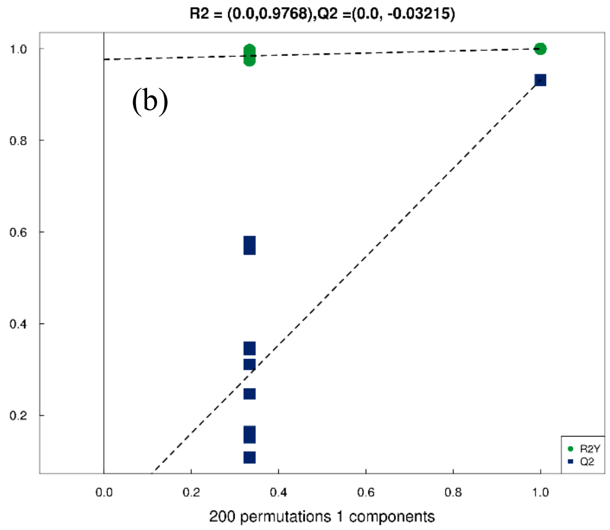
R2 = (0.0,0.9768),Q2 =(0.0, -0.03215)
(b)
1.0
0.8
0.6
0.4
0.2
0.0
0.2
0.4
0.6
0.8
1.0
R2Y
Q2
200 permutations 1 components

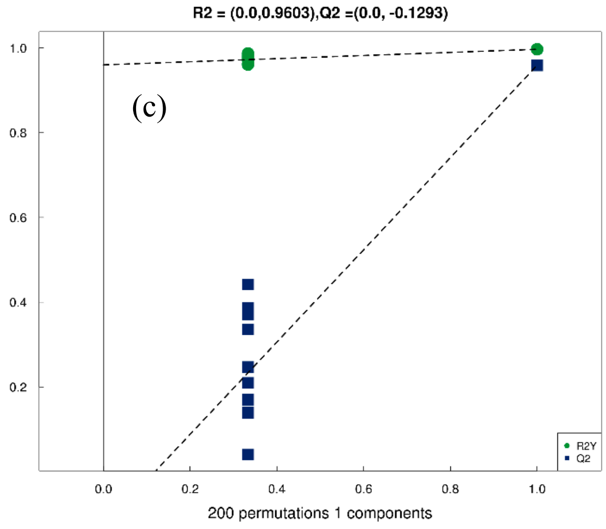
R2 = (0.0,0.9603),Q2 =(0.0, -0.1293)
(c)
1.0
0.8
0.6
0.4
0.2
0.0
0.2
0.4
0.6
0.8
1.0
R2Y
Q2
200 permutations 1 components

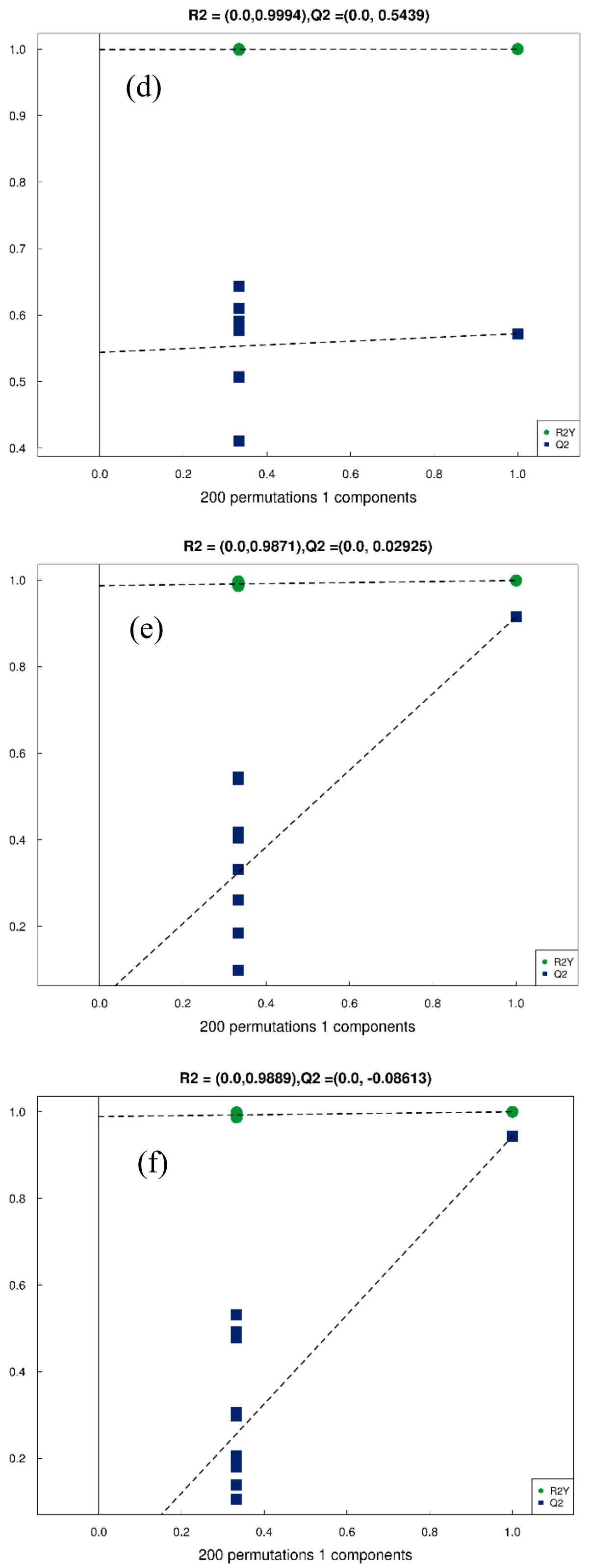

Figure S7. Permutation tests for samples in different erythromycin treatments compared with the control. The 0.001 mg/L (a), 0.75 mg/L (b) and 2.5 mg/L (c) erythromycin treatment was compared to the control, respectively, in electrospray ionization (ESI) negative model; The 0.001 mg/L (d), 0.75 mg/L (e) and 2.5 mg/L(f) erythromycin treatment was compared to the control, respectively, in ESI positive model.

Table S1. Identification of differential metabolites in the negative mode between the treatment groups and control. VIP: variable influence on projection.

Name	VIP	Fold change	p-value	m/z	rt(s)
**Control vs 0.001 mg/L erythromycin treatment**					
2,4,5-trichlorophenol	1.556614581	0.274122274	0.004244194	196.89465	407.679
Capric acid	3.031409968	0.738752621	0.010772363	171.13853	66.2579
Kendomycin	4.387990754	0.722752363	0.011341341	485.28339	43.16735
Dodecyl phosphate	1.19922142	1.56619274	0.012930814	265.14847	120.652
Picloram	1.281336552	0.199264015	0.014003625	194.9473	408.0675
Linolenic acid	1.188905393	2.086620028	0.019015164	277.21756	106.3575
Undecanoic acid	1.312659696	0.768436224	0.034931821	185.15431	66.4525
5s-hydroxy-6e,8z,11z,14z-eicosatetraenoic acid	1.960734262	3.238474107	0.042113765	301.21775	104.3025
**Control vs 0.75 mg/L erythromycin treatment**					
5s-hydroxy-6e,8z,11z,14z-eicosatetraenoic acid	6.874464037	49.80068209	0.000609333	301.21775	104.3025
2,4,5-trichlorophenol	1.198215983	0.007767511	0.000797784	196.89465	407.679
Ganoderic acid h	2.725697745	68.28867189	0.001322689	553.26843	256.611
Leukotriene f4	2.252203331	112.5803779	0.002183321	549.28355	248.631
Pentadecanoic acid	1.245707148	2.050668822	0.00256425	241.21753	107.754
15-hydroxy-5z,8z,11z,13e,17z-eicosapentaenoic acid	2.322087085	20.98867931	0.003156048	317.21255	109.412
Nabilone	2.14113659	22.54299991	0.004689962	247.17055	108.0685
Capric acid	2.450727201	0.601355163	0.004808929	171.13853	66.2579
Undecanoic acid	1.047413821	0.638477093	0.005731049	185.15431	66.4525
Linolenic acid	2.380374984	9.3878195	0.006357915	277.21756	106.3575
Myristic acid	6.448829847	5.087641243	0.007848893	227.2017	108.434
Prostaglandin f2.beta.	1.86535101	11.76572422	0.009098951	317.21243	65.9965
Kendomycin	2.876583538	0.717166955	0.009541576	485.28339	43.16735

1-oleoyl-2-hydroxy-sn-glycero-3-phospho-(1'-rac-glycerol)	1.457839393	18.08023763	0.00973089	509.28943	65.9965
3-glucuronyl-22-ddmp soyasapogenol b	1.33028967	2.366389116	0.010079683	759.43566	65.9251
Stearidonic acid	2.510008212	47.4938797	0.012034433	275.20222	105.8135
Prostaglandin i2	1.179964241	3.279908507	0.016766583	351.21856	250.568
1-palmitoyl-2-hydroxy-sn-glycero-3-phospho-(1'-rac-glycerol)	3.202337603	32.72184539	0.016854435	483.27364	250.244
Isocitric acid	1.160404208	0.325843697	0.025389829	111.00777	843.5375
Prostaglandin a2	1.02496174	3.573340838	0.025822069	315.19708	108.146
Dinor-12-oxophytodienoic acid	1.017841831	3.993408361	0.029024519	263.16546	110.815
Pg 32:2	3.828235319	3.783829132	0.030827312	717.47284	65.94105
Ginsenoside rg5	1.160390125	2.540764533	0.032640006	765.48195	104.5405
Lys-lys	1.174285754	3.622380024	0.036237524	273.18663	106.679
Lauric isopropanolamide	1.627421063	3.268421321	0.043535147	256.23671	106.416
1-hexadecyl lysophosphatidic acid	2.36008796	1.966618636	0.048612939	791.49946	65.76935
**Control vs 2.5 mg/L erythromycin treatment**					
Nabilone	2.209720099	52.2870911	3.86758E-06	247.17055	108.0685
Phosphoric acid	9.703443163	13.15798992	9.97652E-06	96.95906	384.592
Capric acid	2.738681588	0.3371113	0.000475688	171.13853	66.2579
Myristic acid	7.051644569	12.24455678	0.000529257	227.2017	108.434
Prostaglandin f2.beta.	1.301300404	13.06699558	0.0007372	317.21243	65.9965
5s-hydroxy-6e,8z,11z,14z-eicosatetraenoic acid	5.54729822	74.5382058	0.000766702	301.21775	104.3025
Prostaglandin i2	1.658709859	10.81994291	0.000787907	351.21856	250.568
Undecanoic acid	1.289519237	0.316531545	0.00130674	185.15431	66.4525
Pentadecanoic acid	1.201997778	3.72208511	0.001588484	241.21753	107.754
Stearidonic acid	2.768219633	124.0168477	0.00197629	275.20222	105.8135
Lys-lys	1.237458392	7.52263015	0.002027979	273.18663	106.679

Linolenic acid	3.056780052	32.13641276	0.002078829	277.21756	106.3575
Myristoleic acid	1.088344444	28.56826418	0.002328912	225.18587	108.008
15-hydroxy-5z,8z,11z,13e,17z-eicosapentaenoic acid	2.141632486	39.78054184	0.002849685	317.21255	109.412
Fa 16:3+1o	1.244824776	6.99826672	0.003185154	265.18148	66.23735
Methylphosphonic acid	2.333473794	6.21547327	0.00329732	94.97977	218.822
2,4-dichlorobenzoic acid	1.047429686	0.382866477	0.003916365	188.95134	65.76935
N-arachidonoyl-l-alanine	1.523814127	0.115054551	0.004984997	374.2455	64.0623
N-(phosphonomethyl)glycine	1.11432958	4.248325955	0.005567351	149.98498	199.775
Propionic acid	5.090589232	0.485129015	0.005644918	73.02835	67.3162
Progesterone	1.105527415	25.37802825	0.009205128	313.23898	220.377
4-nitrocatechol	1.435637205	34.73982023	0.010151169	154.0173	354.5035
Dodeca-2(e),4(e)-dienoic acid	2.025348	0.519999949	0.010603749	195.13879	64.75815
Kendomycin	3.439119391	0.55771485	0.011618084	485.28339	43.16735
2-hydroxyanthraquinone	1.044084443	0.134290039	0.012591102	223.02819	63.1432
Dinor-12-oxophytodienoic acid	1.542354935	17.26188054	0.012615543	263.16546	110.815
All-trans-4-ketoretinoic acid	1.421850805	9.591623007	0.012949054	269.2129	111.642
[6]-gingerol	1.849695799	1.989956831	0.015358486	293.17642	108.2715
8,11-tridecadienoic acid, 13-(3-pentyl-2-oxiranyl)-, (8z,11z)-	1.721277645	0.525545395	0.018868501	221.15455	65.8255
Probucol	2.134886465	0.504583343	0.01957565	236.1055	65.8255
2'-chloro-2-hydroxy-5-methylbenzophenone	1.096032477	39.22284064	0.025091655	245.04332	362.5755
Octadecanoic acid	4.934615986	0.384928488	0.025831501	283.26461	66.4525
Gentisic acid	1.179170719	36.5979362	0.02959738	152.9952	338.1515
1-hexadecyl lysophosphatidic acid	2.068791665	0.123095182	0.033852067	791.49946	65.76935
Isocitric acid	1.023292712	0.460165527	0.039183661	111.00777	843.5375
Nabilone	2.209720099	52.2870911	3.86758E-06	247.17055	108.0685

Table S2. Identification of differential metabolites in the positive mode between the erythromycin treatment groups and control. VIP: variable influence on projection.

**Name**	**VIP**	**Fold change**	**p-value**	**m/z**	**rt(s)**
**Control vs 0.001 mg/L erythromycin treatment**					
Erythromycin	8.242961801	0.000580068	2.49268E-15	734.46915	273.684
Sclareolide	2.55131892	6.775899076	0.00071181	233.19	245.347
5.alpha.-pregnane-3.alpha.,20.alpha.-diol	1.219492439	26.93377248	0.00124889	285.24229	245.2965
Cefadroxil	3.961122875	0.005664953	0.002433688	158.02838	351.3085
Palmitic acid alkyne	1.541078556	5.699206094	0.005895808	217.19512	244.55
Proscillaridin a	1.805498646	4.918986374	0.00744519	513.30391	243.667
N-acetylhistamine	1.051661761	1.242649926	0.007796719	136.08705	2.04988
Acetylcarnitine	4.189120809	1.631791306	0.008625097	204.12316	358.5455
Ethylenediaminetetraacetic acid	2.300088152	2.181303714	0.009988522	293.09778	385.7235
Ectoine	8.830838203	1.690852763	0.01283989	143.08166	376.582
Hyocholic acid	3.993116622	0.593559734	0.019441057	158.16529	348.268
Monolinolenin (9c,12c,15c)	1.150556464	4.407895685	0.020277503	353.26851	243.2355
Dibutyl phthalate	1.022115411	0.746562701	0.021048	279.15896	486.032
Hexaethylene glycol	2.013100266	2.27570679	0.022371188	283.17502	129.2085
Desogestrel	2.422563445	3.460419828	0.032285997	311.25792	243.762
1-Palmitoylglycerol	1.625727417	2.449827152	0.03326028	313.27354	251.2755
Argatroban	2.781871214	4.149365132	0.036347289	509.2724	245.6555
(cis+trans)-nerodilol	2.681899724	2.65885756	0.038416011	109.07642	85.387
3,4-dimethoxycinnamic acid	1.66084215	2.166878604	0.03859703	167.09278	268.3985
5alpha-androstan-17beta-ol-3-one	1.203595197	27.34482787	0.038678015	215.17951	245.523
**Control vs 0.75 mg/L erythromycin treatment**					
N-palmitoylglycine	1.942631099	30.48261279	4.14194E-06	314.27695	252.7245

1-Palmitoylglycerol	4.420501357	23.74628426	1.00247E-05	313.27354	251.2755
Proscillaridin a	3.573865846	34.64621638	3.17325E-05	513.30391	243.667
Argatroban	7.235599569	44.25377036	0.000184337	509.2724	245.6555
D-(-)-penicillamine	2.257438693	19.15547642	0.000862787	149.13258	245.246
Caldine	1.064150554	1.266119226	0.001094732	115.12334	296.45
1,2-dilinolenoyl-sn-glycero-3-phosphoethanolamine	2.725578316	3.448546946	0.002531318	736.49164	259.581
Cefadroxil	2.629952252	0.024344253	0.002616794	158.02838	351.3085
Pristimerin	3.386895549	128.9689133	0.00280811	487.28984	245.162
Ala-Ala	1.366433053	9.408241062	0.003027546	161.09236	382.5095
DL-proline	1.421777781	1.765591613	0.003181536	116.07093	366.871
Methysticin	1.165198075	7.454126976	0.003290275	275.07363	372.0855
1-Stearoyl-sn-glycerol 3-phosphocholine	1.732018101	12.45445356	0.004028759	568.33985	277.461
1-oleoyl-2-palmitoyl-rac-glycerol	1.571499915	12.16579657	0.006441658	339.28928	251.18
Gamma-l-glutamyl-l-glutamic acid	9.169183471	11.90882449	0.006506335	277.08948	372.096
5.alpha.-pregnane-3.alpha.,20.alpha.-diol	2.63999919	1.169836933	0.006920601	285.24229	245.2965
Monolinolenin (9c,12c,15c)	3.202065237	60.99742573	0.006948583	353.26851	243.2355
Glycerol 1-myristate	2.034376105	39.04098005	0.007248765	303.25288	245.246
1,2-diarachidonoyl-sn-glycero-3-phosphocholine	1.009321281	3.32500918	0.007932649	830.56895	256.49
Lpc 18:2	3.366951099	12.3509158	0.008568805	520.34034	279.556
1-Palmitoyl-sn-glycero-3-phosphocholine	5.161604538	30.7153849	0.009628812	518.32451	280.3545
PC (16:0/16:0)	2.278949347	3.01358978	0.009724868	800.52237	256.96
10-nitrooleic acid	2.736865551	48.98455909	0.009813604	310.24572	244.653
Desogestrel	5.850092056	30.97750345	0.010029983	311.25792	243.762
2-linoleoyl-1-palmitoyl-sn-glycero-3-phosphoethanolamine	1.04210309	3.602355849	0.010190354	575.50371	226.7485
5alpha-androstan-17beta-ol-3-one	4.03814699	1.631787787	0.011302928	215.17951	245.523

1,2-di-(9z,12z,15z-octadecatrienoyl)-sn-glycero-3-phosphocholine	7.707950969	3.278479675	0.011929137	778.53876	257.759
Sclareolide	6.537987358	91.11549334	0.012233842	233.19	245.347
Patchouli alcohol	2.022254504	18.23150441	0.012403723	135.11695	244.9955
1,2-dipalmitoleoyl-sn-glycero-3-phosphocholine	11.0084937	3.836021337	0.013066049	730.53904	259.4925
Acridine orange	2.052998281	0.344346476	0.013352589	266.17259	245.865
Thiamine	2.23631243	5.140534028	0.014021048	265.11134	501.787
1,2-dipalmitoleoyl-sn-glycero-3-phosphoethanolamine	3.983526578	3.360604672	0.01410188	688.49172	261.251
Dipalmitolein (9c)	3.733271352	3.266885078	0.014662732	547.47253	227.6425
N-acetyl-d-glucosamine	4.56129744	3.618056044	0.014702197	138.05511	324.4285
Reserpine	5.225179616	12.97079471	0.014734737	609.27093	233.292
1-palmitoyl-2-linoleoyl-rac-glycerol	1.545919732	4.773562836	0.015720198	313.27353	225.496
Heptadecasphinganine	4.125433424	1.919669364	0.015738587	288.28969	224.1875
Jaeschkeanadiol	1.575520825	9.94775891	0.016453421	261.18481	107.174
Hydrocortisone 17-acetate	2.787287049	8.43579684	0.017095562	297.20591	107.183
Vecuronium	3.367199521	2.992248187	0.017382444	557.4204	109.2325
Thioetheramide-PC	5.432274962	3.166748779	0.017961644	802.53869	257.3115
Nigerose	1.059627603	3.74476021	0.018320179	365.10528	392.997
1-oleoyl-sn-glycero-3-phosphoethanolamine	2.15580185	11.29894516	0.020748168	480.30862	281.87
Ectoine	19.36315102	7.966825851	0.021324818	143.08166	376.582
Fucoxanthin	8.433459	2.761297755	0.021931049	641.42028	63.7373
Phosphocholine	3.388307388	2.91566543	0.025484996	184.07343	258.766
Suberic acid	3.411992095	6.426575287	0.025652084	139.08667	103.13
Aldosterone	1.003122445	1.381059673	0.026244278	315.19529	107.3955
5-hydroxyvalproic acid	1.229780664	5.528082756	0.026441062	143.11312	376.823
Citrazinic acid	1.678343947	4.603089255	0.02780976	156.03257	351.393

1-.beta.-d-arabinofuranosyluracil 5'-monophosphate	1.750422195	0.780018471	0.027990548	390.99376	484.217
Dicyclohexylamine	9.731836955	0.707317082	0.029379839	100.11259	312.7535
PC (20:5(5Z,8Z,11Z,14Z,17Z)/20:5(5Z,8Z,11Z,14Z,17Z))	1.785447402	2.516891994	0.032562931	826.5382	255.564
1,2-dioleoyl-sn-glycero-3-phosphatidylcholine	1.755031497	2.427862666	0.033266162	852.5544	254.56
Choline	5.897542102	2.219363623	0.037089045	104.10744	435.4715
Guanidoacetic acid	1.877553308	2.491063622	0.037535606	118.06543	244.653
Aloeemodin	2.962199469	9.249558526	0.038085272	607.25535	57.9812
Acetylcarnitine	6.139048575	4.137908013	0.039068576	204.12316	358.5455
Glycerophosphocholine	2.839113142	3.210168912	0.039343731	258.11005	397.509
Arachidonoyl-n,n-dimethylamide	1.249232116	1.799830901	0.042943659	332.31573	242.361
S-methyl-5'-thioadenosine	1.381509052	1.709881049	0.047912995	298.09672	249.642
Palmitic acid alkyne	4.607527531	108.9362895	0.048340661	217.19512	244.55
DL-Glutamic acid	1.113507878	8.145800948	0.049133549	148.04122	394.863
**Control vs 2.5 mg/L erythromycin treatment**					
Thiamine	3.306202292	13.2393561	4.45338E-05	265.11134	501.787
Adenine	4.764855079	5.74842237	4.70312E-05	136.06192	288.517
Benzamide, n-1h-pyrrolo[2,3-c]pyridin-5-yl-	1.556901395	4.822977112	6.24563E-05	238.08221	298.533
Methysticin	1.705159701	20.60483106	7.46524E-05	275.07363	372.0855
Isobutyryl-l-carnitine	1.030845939	5.120266488	9.98303E-05	232.1543	328.5695
4-methylmethcathinone	1.354886249	0.466390351	0.000113455	178.12267	276.3005
Glutamic acid	2.509287303	6.15704186	0.000136152	148.0605	383.016
Hydroquinone	2.823661216	19.91781971	0.000154981	111.04445	232.203
DL-asparagine	1.493684915	20.97607959	0.000175182	133.06124	389.221
Leu-Val	1.351040787	28.45397773	0.000206957	231.17019	293.4565
Phosphocholine	3.64323682	4.079447601	0.000234408	184.07343	258.766

1,2-dipalmitoleoyl-sn-glycero-3-phosphocholine	10.43346526	4.696749457	0.000321824	730.53904	259.4925
G-guanidinobutyrate	1.284670988	3.016872518	0.000371548	146.09247	387.811
1,2-di-(9z,12z,15z-octadecatrienoyl)-sn-glycero-3-phosphocholine	9.10157839	5.465243322	0.000433029	778.53876	257.759
1,2-diarachidonoyl-sn-glycero-3-phosphocholine	1.267696026	6.201590571	0.000462225	830.56895	256.49
Suberic acid	3.69718899	9.734894337	0.000469583	139.08667	103.13
D-glutamine	7.79553507	69.16412798	0.000597305	147.0765	388.655
Sucrose	1.19960412	139.813799	0.000659381	381.06268	371.992
3,4-dimethoxycinnamic acid	4.333002682	24.93756804	0.000669397	167.09278	268.3985
Tyramine	3.29635267	4.613613783	0.000689316	138.10265	274.9335
Isomangiferin	1.03755583	32.6931538	0.000739254	423.11001	371.465
1-Aminocyclopropanecarboxylic acid	3.719672101	10.81171556	0.000780929	84.04502	387.562
4-hydroxy-l-isoleucine	1.121448443	4.787004657	0.000839188	102.09181	491.691
N-acetyl-l-phenylalanine	1.268272199	3.460799578	0.00086393	120.08106	330.348
Fucoxanthin	10.69418406	4.873594571	0.000941264	641.42028	63.7373
1,2-dilinolenoyl-sn-glycero-3-phosphoethanolamine	1.995186032	3.130152935	0.001145356	736.49164	259.581
DL-arginine	9.478371904	13.03790573	0.001233577	175.119	546.382
5.alpha.-pregnane-3.alpha.,20.alpha.-diol	1.936069197	1.423968935	0.00123392	285.24229	245.2965
PC (16:0/16:0)	2.597361737	4.725688711	0.001248254	800.52237	256.96
Gamma-l-glutamyl-l-glutamic acid	20.01137595	74.8048619	0.001274285	277.08948	372.096
Acetylcarnitine	6.788236169	6.24806499	0.001301927	204.12316	358.5455
Ala-Ala	1.460137071	14.95568854	0.00131644	161.09236	382.5095
Phendimetrazine	1.601961311	0.772551032	0.001434021	192.13835	299.0395
Arginine	2.519390861	4.525595859	0.001436609	175.11898	577.4265
Glycerol 1-myristate	1.004341655	14.54101575	0.00144288	303.25288	245.246
Thioetheramide-PC	5.441665537	4.101715256	0.001676252	802.53869	257.3115

15-deoxy-delta-12,14-pgj2	5.685174936	3058.104029	0.002077973	317.2111	36.1811
N8-acetylspermidine	1.128036734	0.884830596	0.002136703	171.14925	297.477
1-palmitoyl-2-linoleoyl-rac-glycerol	1.299949592	4.862939967	0.002208651	313.27353	225.496
1-Palmitoylglycerol	4.447610841	34.91262989	0.002252653	313.27354	251.2755
Lipoxin a4	5.084595708	1143.895984	0.002262565	299.20053	35.89295
N-methyl-d-glucamine	3.997495116	0.728146989	0.002463011	178.12276	300.6845
Gly-Leu-Arg	1.133961172	127.8227533	0.002497336	345.22422	390.716
Cycloheximide	1.463866924	22.26920515	0.002516398	246.15594	418.2795
Benzamide, n-[2-(dimethylamino)ethyl]-n-methyl-4-[[[[4-[4-(4-morpholinyl)-7-(2,2,2-trifluoroethyl)-7h-pyrrolo[2,3-d]pyrimidin-2-yl]phenyl]amino]carbonyl]amino]-	1.284022865	28.61098211	0.00253043	625.2657	244.9555
Pheophytin a	1.575116738	0.583068048	0.002595348	871.57348	64.2553
Glycerol	1.313214007	91.54939768	0.002625973	93.05517	371.992
Phe-leu	1.640751791	39.49596312	0.002667504	279.17003	271.5235
DL-Glutamic acid	1.605546691	21.50777396	0.002854344	148.04122	394.863
Met-Leu	1.16966814	36.2579269	0.00300128	263.14234	286.61
Desogestrel	2.369870182	8.101790358	0.003170682	311.25792	243.762
Dimethametryn	1.383799511	9.593128876	0.003434227	256.14428	63.92875
Pseudoerythromycin a enol ether	2.718709634	93.44436221	0.003467141	558.36375	273.389
D-(+)-galactose	1.234395038	51.52132314	0.00354937	163.06019	372.128
1-oleoyl-sn-glycero-3-phosphoethanolamine	1.048386509	4.610603433	0.003570404	480.30862	281.87
All-trans-4-hydroxyretinoic acid	1.845543993	745.8518371	0.003790705	281.1899	36.2909
Oxandrolone	3.304087064	0.23313082	0.004069689	307.22669	108.255
Acetoveratrone	1.300961555	6.698026882	0.004180647	139.09789	353.286
Leucylleucine	1.675727009	58.97215437	0.004244135	245.18591	284.025

Vecuronium	2.271851603	0.090016351	0.004660975	557.4204	109.2325
1,2-dioleoyl-sn-glycero-3-phosphatidylcholine	1.76657041	3.066381549	0.004666323	852.5544	254.56
N-acetyl-d-glucosamine	18.60481571	57.61010138	0.00471152	138.05511	324.4285
Cefadroxil	2.248086998	0.154184515	0.004760394	158.02838	351.3085
PC (20:5(5Z,8Z,11Z,14Z,17Z)/20:5(5Z,8Z,11Z,14Z,17Z))	2.044625843	3.781967604	0.004852574	826.5382	255.564
2-imino-4-methylpiperidine	1.102372285	0.80420768	0.005081225	113.10768	330.955
Hydrocortisone 17-acetate	1.250034066	3.370901732	0.005376893	297.20591	107.183
Aldosterone	1.31877716	1.783141821	0.005647039	315.19529	107.3955
Erythromycin	15.05868374	13.03470867	0.006474049	734.46915	273.684
Meperidine	1.10130223	7.589114289	0.006574332	174.13495	551.9205
Anilazine	1.439547572	29.83224099	0.006796985	178.01452	351.302
Phytosphingosine	1.647721829	0.596149547	0.007045699	318.30018	232.166
Citrazinic acid	3.928748636	27.19014091	0.007185413	156.03257	351.393
Acridine orange	2.074780681	0.264548567	0.007493869	266.17259	245.865
2-[[7-hydroxy-1-(4-hydroxy-3,5-dimethoxyphenyl)-3-(hydroxymethyl)-6,8-dimethoxy-1,2,3,4-tetrahydronaphthalen-2-yl]methoxy]-6-(hydroxymethyl)oxane-3,4,5-triol	1.298150089	20.01409023	0.007608978	605.23972	265.1595
Sclareolide	1.481243195	8.019415037	0.007752595	233.19	245.347
DL-proline	2.293225754	3.852187343	0.007875317	116.07093	366.871
Cyclopentolate	1.468652683	21.72452299	0.008320917	274.18716	389.982
2s-amino-4-phosphonobutyric acid	1.813767662	29.61740974	0.008459548	138.02204	351.3155
Dipalmitolein (9c)	2.267270824	2.489972884	0.008969701	547.47253	227.6425
Isopropalin	1.149749769	0.220402956	0.009384488	210.11006	302.181
N-acetyl-o-fluoro-dl-phenylalanine	2.52177359	18.80951931	0.010086417	226.0838	399.954
Miglitol	1.125186478	0.518375443	0.010206462	208.11799	303.2095

Caldine	1.365331263	0.879932543	0.010478917	115.12334	296.45
N-demethylerythromycin	2.696531735	56.14462566	0.01079045	720.45338	287.662
(+)-aphidicolin	2.091069599	0.252915037	0.011332329	285.24228	109.254
1-Palmitoyl-sn-glycero-3-phosphocholine	5.560329801	49.75435476	0.012205717	518.32451	280.3545
L-carnitine	4.771679632	6.696068079	0.012813017	162.11254	384.981
Fumagillin	1.398629936	18.70704811	0.012948224	265.17967	107.6025
Trigonelline	4.583447266	74.63128041	0.013553475	160.037	324.1705
1-myristoyl-sn-glycero-3-phosphocholine	1.244693359	46.12774612	0.013696008	468.30875	282.9435
Atorvastatin	2.280078501	139.6329869	0.014079359	541.26223	270.6295
4-[5-[[4-[5-[acetyl(hydroxy)amino]pentylamino]-4-oxobutanoyl]-hydroxyamino]pentylamino]-4-oxobutanoic acid	1.006784724	21.67909418	0.014080683	478.29325	282.8405
L-leucyl-l-proline	1.878176582	104.7232736	0.014237443	229.15878	268.4055
1-Stearoyl-sn-glycerol 3-phosphocholine	2.842486299	44.54355416	0.014297501	568.33985	277.461
Glycerophosphocholine	10.23316522	40.87515528	0.015403729	258.11005	397.509
Lpc 18:2	4.923732163	35.33937001	0.017045555	520.34034	279.556
Hydroxyflutamide	1.414714059	12.34622545	0.017868177	275.07359	319.546
Scopolamine	1.236176657	0.493841633	0.018346621	138.10268	1.5808
(2r,3s,4s,5r,6r)-5-[(2s,3r,4r)-3,4-dihydroxy-4-(hydroxymethyl)oxolan-2-yl]oxy-2-(hydroxymethyl)-6-(2-phenylethoxy)oxane-3,4-diol	3.809907087	707.9245603	0.019144268	439.14205	415.391
Deoxyadenosine	1.524827493	24.71287995	0.019918584	252.10883	278.827
Phlorizin	1.531472986	133.567009	0.019976838	437.12676	373.8625
Tris(hydroxymethyl)aminomethane	12.28449311	0.521161275	0.020497862	122.08143	899.482
Ectoine	7.229468036	2.691239571	0.021168107	143.08166	376.582
Proscillaridin a	1.214232735	7.275405103	0.021942884	513.30391	243.667

Isopimpinellin	1.178812398	50.00595158	0.022084787	269.03953	359.9485
Convallatoxin	1.125894402	60.55255404	0.023067396	589.24463	254.687
2-hexen-1-ol, (z)-	1.356915483	1.524910326	0.023794636	83.0862	313.254
Putrescine	1.261810747	0.457093563	0.024528905	89.10791	349.7075
.gamma.-aminobutyric acid	4.531361888	12.43939925	0.025027118	104.07102	459.2635
5-(2-hydroxyethyl)-4-methylthiazole	1.216946803	12.39243576	0.025677752	144.04781	500.895
Aniline	2.876299433	41.38504538	0.028512198	94.06567	324.454
N-palmitoylglycine	2.030079732	49.51320039	0.029874182	314.27695	252.7245
2-palmitoyl-rac-glycerol	1.297850474	1.853447155	0.035019821	313.27339	65.5423
S-methyl-5'-thioadenosine	1.204354713	0.664727677	0.037887913	298.09672	249.642
Guanidoacetic acid	1.005887242	0.716456707	0.041703679	118.06543	244.653
L-Glutamate	1.557177657	1.825508515	0.041949052	130.05007	296.487
1,2-dipalmitoleoyl-sn-glycero-3-phosphoethanolamine	2.21198152	2.430224631	0.042009723	688.49172	261.251
Cortodoxone	1.794724898	14.6402428	0.043615968	293.21093	108.353
Pentapropylene glycol	2.890821937	0.575414777	0.044926652	309.2269	64.17515
Choline	7.650120084	4.380793118	0.045710041	104.10744	435.4715
Ile-Arg	2.03228593	62.09463643	0.045973274	288.20291	370.2785
Memantine	1.480166316	0.394877948	0.046940733	180.17472	246.2965
Ethylenediaminetetraacetic acid	1.750607359	3.582771389	0.047354847	293.09778	385.7235
Reserpine	4.111883478	12.54113419	0.047913211	609.27093	233.292
Thiamine	3.306202292	13.2393561	4.45338E-05	265.11134	501.787
Adenine	4.764855079	5.74842237	4.70312E-05	136.06192	288.517
Benzamide, n-1h-pyrrolo[2,3-c]pyridin-5-yl-	1.556901395	4.822977112	6.24563E-05	238.08221	298.533
Methysticin	1.705159701	20.60483106	7.46524E-05	275.07363	372.0855
Isobutyryl-l-carnitine	1.030845939	5.120266488	9.98303E-05	232.1543	328.5695

4-methylmethcathinone	1.354886249	0.466390351	0.000113455	178.12267	276.3005
Glutamic acid	2.509287303	6.15704186	0.000136152	148.0605	383.016
Hydroquinone	2.823661216	19.91781971	0.000154981	111.04445	232.203
DL-asparagine	1.493684915	20.97607959	0.000175182	133.06124	389.221
Leu-Val	1.351040787	28.45397773	0.000206957	231.17019	293.4565
Phosphocholine	3.64323682	4.079447601	0.000234408	184.07343	258.766
1,2-dipalmitoleoyl-sn-glycero-3-phosphocholine	10.43346526	4.696749457	0.000321824	730.53904	259.4925
G-guanidinobutyrate	1.284670988	3.016872518	0.000371548	146.09247	387.811
1,2-di-(9z,12z,15z-octadecatrienoyl)-sn-glycero-3-phosphocholine	9.10157839	5.465243322	0.000433029	778.53876	257.759
1,2-diarachidonoyl-sn-glycero-3-phosphocholine	1.267696026	6.201590571	0.000462225	830.56895	256.49
Suberic acid	3.69718899	9.734894337	0.000469583	139.08667	103.13
D-glutamine	7.79553507	69.16412798	0.000597305	147.0765	388.655
Sucrose	1.19960412	139.813799	0.000659381	381.06268	371.992
3,4-dimethoxycinnamic acid	4.333002682	24.93756804	0.000669397	167.09278	268.3985
Tyramine	3.29635267	4.613613783	0.000689316	138.10265	274.9335

Table S3. Integrated analyses of differentially accumulated metabolites and genes in *T. weissflogii* treated with erythromycin.

**Metabolics Compound ID**	**Protein ID**	**Metabolics Description**	**EC**	**KO**	**Protein Description**	**Gene**
**Control vs 0.001 mg/L erythromycin treatment**						
C05135	TRINITY_DN1976_c0_g1	N-acetylhistamine	2.3.1.-	K13511	Monolysocardiolipin acyltransferase	*taz*
C05135	TRINITY_DN3136_c0_g1	N-acetylhistamine	2.3.1.-	K14521	N-acetyltransferase 10	*nat10*
**Control vs 0.75 mg/L erythromycin treatment**						
C07101	TRINITY_DN2223_c0_g1	2,4,5-trichlorophenol	1.14.13.-	K06126	Ubiquinone biosynthesis monooxygenase Coq6	*coq6*
C00311	TRINITY_DN8067_c0_g1	Isocitric acid	4.2.1.3	K01681	Aconitate hydratase	*aco*
C00311	TRINITY_DN2000_c0_g1	Isocitric acid	4.2.1.3	K01681	Aconitate hydratase	*aco*
C00311	TRINITY_DN951_c2_g1	Isocitric acid	4.1.3.1	K01637	Isocitrate lyase	*aceA*
C00378	TRINITY_DN4826_c0_g1	Thiamine	3.1.3.2	K14410	Lysosomal acid phosphatase	*acp2*
C00378	TRINITY_DN37805_c0_g1	Thiamine	3.1.3.2	K14379	Tartrate-resistant acid phosphatase type 5	*acp5*
C16650	TRINITY_DN2223_c0_g1	5-hydroxyvalproic acid	1.14.13.-	K06126	Ubiquinone biosynthesis monooxygenase Coq6	*coq6*
C00114	TRINITY_DN303_c0_g1	Choline	2.6.-.-	K05290	GPI-anchor transamidase subunit K	*pigk*
C00114	TRINITY_DN220_c0_g1	Choline	3.1.4.46	K01126	Glycerophosphoryl diester phosphodiesterase	*glpQ*

C00670	TRINITY_DN220_c0_g1	Glycerophosphocholine	3.1.4.46	K01126	Glycerophosphoryl diester phosphodiesterase	*glpQ*
C00670	TRINITY_DN43341_c0_g1	Glycerophosphocholine	3.1.1.5	K06130	Lysophospholipase II	*lypla2*
C00170	TRINITY_DN2211_c0_g1	S-methyl-5'-thioadenosine	4.4.1.14	K01762	1-aminocyclopropane-1-carboxylate synthase	*acs*
C00170	TRINITY_DN174_c0_g1	S-methyl-5'-thioadenosine	2.5.1.16	K00797	Spermidine synthase	*speE*
C00170	TRINITY_DN1400_c0_g1	S-methyl-5'-thioadenosine	2.5.1.16	K00797	Spermidine synthase	*speE*
C00170	TRINITY_DN1849_c0_g1	S-methyl-5'-thioadenosine	2.5.1.79	K18787	Thermospermine synthase	*acl5*
C00170	TRINITY_DN1825_c0_g1	S-methyl-5'-thioadenosine	2.5.1.79	K18787	Thermospermine synthase	*acl5*
**Control vs 2.5 mg/L erythromycin treatment**						
C00009	TRINITY_DN809_c0_g1	Phosphoric acid	2.7.9.3	K01008	Selenide, water dikinase	*selD*
C00009	TRINITY_DN3740_c0_g1	Phosphoric acid	6.3.2.11	K14755	Carnosine synthase	*crns1*
C00009	TRINITY_DN2917_c0_g1	Phosphoric acid	4.1.1.31	K01595	Phosphoenolpyruvate carboxylase	*ppc*
C00009	TRINITY_DN344_c6_g1	Phosphoric acid	4.3.3.6	K06215	Pyridoxal 5'-phosphate synthase pdxS subunit	*pdxS*

C00009	TRINITY_DN549_c2_g1	Phosphoric acid	3.6.5.4	K03106	Signal recognition particle subunit SRP54	*srp54*
C00009	TRINITY_DN21_c0_g1	Phosphoric acid	3.6.5.4	K03106	Signal recognition particle subunit SRP54	*srp54*
C00009	TRINITY_DN2634_c0_g1	Phosphoric acid	6.3.4.2	K01937	CTP synthase	*pyrG*
C00009	TRINITY_DN10971_c0_g1	Phosphoric acid	3.6.1.-	K14539	Large subunit GTPase 1	*lsg1*
C00009	TRINITY_DN1586_c0_g1	Phosphoric acid	3.6.1.-	K01519	XTP/dITP diphosphohydrolase	*rdgB*
C00009	TRINITY_DN42245_c0_g1	Phosphoric acid	3.6.1.-	K14539	Large subunit GTPase 1	*lsg1*
C00009	TRINITY_DN200_c1_g1	Phosphoric acid	2.7.9.2	K01007	Pyruvate, water dikinase	*pps*
C00009	TRINITY_DN17570_c0_g1	Phosphoric acid	6.4.1.3	K01965	Propionyl-CoA carboxylase alpha chain	*pcca*
C00009	TRINITY_DN1303_c0_g1	Phosphoric acid	6.3.5.3	K01952	Phosphoribosylformylglycinamidine synthase	*pfas*
C00009	TRINITY_DN31967_c0_g1	Phosphoric acid	3.1.3.27	K01094	Phosphatidylglycerolphosphatase GEP4	*gep4*
C00009	TRINITY_DN5593_c0_g1	Phosphoric acid	3.6.1.6	K12304	Soluble calcium-activated nucleotidase 1	*cant1*
C00009	TRINITY_DN602_c0_g1	Phosphoric acid	3.1.3.36	K20278	Inositol polyphosphate 5-phosphatase INPP5E	*inpp5e*
C00009	TRINITY_DN3200_c0_g1	Phosphoric acid	3.1.3.36	K01099	inositol polyphosphate 5-phosphatase INPP5B/F	*inpp5b_f*
C00009	TRINITY_DN3057_c0_g1	Phosphoric acid	3.2.1.23	K01190	Beta-galactosidase	*lacZ*
C00009	TRINITY_DN1468_c0_g1	Phosphoric acid	3.6.1.52	K07766	Diphosphoinositol-polyphosphate diphosphatase	*E3.6.1.52*
C00009	TRINITY_DN3649_c0_g1	Phosphoric acid	6.3.4.21	K00763	Nicotinate phosphoribosyltransferase	*pncB*
C00009	TRINITY_DN495_c0_g2	Phosphoric acid	6.3.1.2	K01915	Glutamine synthetase	*glnA*

C00009	TRINITY_DN495_c0_g1	Phosphoric acid	6.3.1.2	K01915	Glutamine synthetase	*glnA*
C00009	TRINITY_DN1550_c0_g1	Phosphoric acid	6.3.1.2	K01915	Glutamine synthetase	*glnA*
C00009	TRINITY_DN111_c1_g1	Phosphoric acid	6.3.1.2	K01915	Glutamine synthetase	*glnA*
C00009	TRINITY_DN3925_c0_g1	Phosphoric acid	6.3.1.2	K01915	Glutamine synthetase	*glnA*
C00009	TRINITY_DN876_c0_g1	Phosphoric acid	3.6.1.1	K01507	Inorganic pyrophosphatase	*ppa*
C00009	TRINITY_DN14230_c1_g1	Phosphoric acid	3.6.1.1	K01507	Inorganic pyrophosphatase	*ppa*
C00009	TRINITY_DN215_c0_g1	Phosphoric acid	2.9.1.2	K03341	O-phospho-L-seryl-tRNASec:L-selenocysteinyl-tRNA synthase	*sepsecs*
C00009	TRINITY_DN1895_c0_g1	Phosphoric acid	6.3.2.2	K11204	Glutamate--cysteine ligase catalytic subunit	*gclc*
C00009	TRINITY_DN8437_c0_g1	Phosphoric acid	4.2.3.4	K01735	3-dehydroquinate synthase	*aroB*
C00009	TRINITY_DN134_c0_g1	Phosphoric acid	6.3.3.2	K01934	5-formyltetrahydrofolate cyclo-ligase	*mthfs*
C00009	TRINITY_DN314_c0_g1	Phosphoric acid	2.5.1.78	K00794	6,7-dimethyl-8-ribityllumazine synthase	*ribH*
C00009	TRINITY_DN36010_c0_g1	Phosphoric acid	3.1.3.48	K18041	Protein tyrosine phosphatase type IVA	*ptp4a*
C00009	TRINITY_DN143_c0_g5	Phosphoric acid	3.1.3.48	K18041	Protein tyrosine phosphatase type IVA	*ptp4a*
C00009	TRINITY_DN1513_c0_g1	Phosphoric acid	6.3.4.13	K01945	Phosphoribosylamine---glycine ligase	*purD*
C00009	TRINITY_DN2650_c0_g1	Phosphoric acid	1.2.1.38	K00145	N-acetyl-gamma-glutamyl-phosphate reductase	*argC*
C00009	TRINITY_DN2231_c0_g1	Phosphoric acid	4.2.3.5	K01736	Chorismate synthase	*aroC*

C00009	TRINITY_DN2076_c0_g1	Phosphoric acid	3.1.3.25	K01092	Myo-inositol-1(or 4)-monophosphatase	*impa*
C00009	TRINITY_DN465_c0_g1	Phosphoric acid	3.1.3.25	K01092	Myo-inositol-1(or 4)-monophosphatase	*impa*
C00009	TRINITY_DN3_c0_g1	Phosphoric acid	3.1.3.11	K03841	Fructose-1,6-bisphosphatase I	*fbp*
C00009	TRINITY_DN238_c0_g1	Phosphoric acid	3.1.3.11	K03841	Fructose-1,6-bisphosphatase I	*fbp*
C00009	TRINITY_DN1206_c0_g1	Phosphoric acid	6.4.1.1	K01958	Pyruvate carboxylase	*pc*
C00009	TRINITY_DN2313_c0_g1	Phosphoric acid	6.4.1.1	K01958	Pyruvate carboxylase	*pc*
C00009	TRINITY_DN5359_c0_g1	Phosphoric acid	1.2.1.11	K00133	Aspartate-semialdehyde dehydrogenase	*asd*
C00009	TRINITY_DN2781_c0_g1	Phosphoric acid	3.1.3.3	K01079	Phosphoserine phosphatase	*serB*
C00009	TRINITY_DN1953_c0_g1	Phosphoric acid	6.6.1.1	K03404	Magnesium chelatase subunit D	*chlD*
C00009	TRINITY_DN1034_c0_g2	Phosphoric acid	6.6.1.1	K03403	Magnesium chelatase subunit H	*chlH*
C00009	TRINITY_DN692_c0_g1	Phosphoric acid	6.6.1.1	K03404	Magnesium chelatase subunit D	*chlD*
C00009	TRINITY_DN61_c0_g3	Phosphoric acid	6.6.1.1	K03403	Magnesium chelatase subunit H	*chlH*
C00009	TRINITY_DN189_c1_g1	Phosphoric acid	6.6.1.1	K03403	Magnesium chelatase subunit H	*chlH*
C00009	TRINITY_DN2440_c0_g1	Phosphoric acid	3.1.3.-	K18551	Pyrimidine and pyridine-specific 5'-nucleotidase	*sdt1*
C00009	TRINITY_DN3892_c0_g1	Phosphoric acid	4.2.3.1	K01733	Threonine synthase	*thrC*
C00009	TRINITY_DN3414_c0_g1	Phosphoric acid	4.2.3.2	K14286	Ethanolamine-phosphate phospho-lyase	*agxt2l1*
C00009	TRINITY_DN2142_c0_g1	Phosphoric acid	2.5.1.19	K00800	3-phosphoshikimate 1-carboxyvinyltransferase	*aroA*

C00009	TRINITY_DN314_c3_g1	Phosphoric acid	3.1.3.37	K01100	Sedoheptulose-bisphosphatase	*E3.1.3.37*
C00009	TRINITY_DN20102_c0_g1	Phosphoric acid	1.2.1.12	K00134	Glyceraldehyde 3-phosphate dehydrogenase	*gapdh*
C00009	TRINITY_DN2695_c0_g1	Phosphoric acid	1.2.1.12	K00134	Glyceraldehyde 3-phosphate dehydrogenase	*gapdh*
C00009	TRINITY_DN998_c0_g1	Phosphoric acid	1.2.1.12	K00134	Glyceraldehyde 3-phosphate dehydrogenase	*gapdh*
C00009	TRINITY_DN562_c1_g1	Phosphoric acid	1.2.1.12	K00134	Glyceraldehyde 3-phosphate dehydrogenase	*gapdh*
C00009	TRINITY_DN2695_c0_g2	Phosphoric acid	1.2.1.12	K00134	Glyceraldehyde 3-phosphate dehydrogenase	*gapdh*
C00009	TRINITY_DN173_c2_g1	Phosphoric acid	6.3.4.3	K01938	Formate--tetrahydrofolate ligase	*fhs*
C00009	TRINITY_DN487_c0_g1	Phosphoric acid	3.1.3.16	K06269	Serine/threonine-protein phosphatase PP1 catalytic subunit	*ppp1c*
C00009	TRINITY_DN2151_c0_g1	Phosphoric acid	3.1.3.16	K14497	Protein phosphatase 2C	*pp2c*
C00009	TRINITY_DN1945_c0_g1	Phosphoric acid	3.1.3.16	K15498	Serine/threonine-protein phosphatase 6 catalytic subunit	*ppp6c*
C00009	TRINITY_DN402_c0_g2	Phosphoric acid	3.1.3.16	K04382	Serine/threonine-protein phosphatase 2A catalytic subunit	*ppp2c*
C00009	TRINITY_DN2700_c0_g1	Phosphoric acid	3.1.3.16	K17618	Ubiquitin-like domain-containing CTD phosphatase 1	*ublcp1*
C00009	TRINITY_DN1687_c0_g1	Phosphoric acid	3.1.3.16	K04460	Serine/threonine-protein phosphatase 5	*ppp5c*

C00009	TRINITY_DN1733_c0_g1	Phosphoric acid	3.1.3.16	K15637	Serine/threonine-protein phosphatase PGAM5	*pgam5*
C00009	TRINITY_DN35702_c0_g1	Phosphoric acid	3.1.3.16	K17499	Protein phosphatase 1G	*ppm1g*
C00009	TRINITY_DN4596_c0_g1	Phosphoric acid	3.1.3.16	K06269	Serine/threonine-protein phosphatase PP1 catalytic subunit	*ppp1c*
C00009	TRINITY_DN25174_c0_g2	Phosphoric acid	3.1.3.16	K17618	Ubiquitin-like domain-containing CTD phosphatase 1	*ublcp1*
C00009	TRINITY_DN1743_c0_g1	Phosphoric acid	3.1.3.16	K17508	Protein phosphatase PTC7	*ptc7*
C00009	TRINITY_DN4364_c0_g1	Phosphoric acid	3.6.1.5	K01510	Apyrase	*entpd1_3_8*
C00009	TRINITY_DN3700_c0_g1	Phosphoric acid	2.7.9.1	K01006	Pyruvate, orthophosphate dikinase	*ppdK*
C00009	TRINITY_DN859_c0_g1	Phosphoric acid	2.7.9.1	K01006	Pyruvate, orthophosphate dikinase	*ppdK*
C00009	TRINITY_DN4035_c0_g1	Phosphoric acid	2.7.9.1	K01006	Pyruvate, orthophosphate dikinase	*ppdK*
C00009	TRINITY_DN2639_c0_g1	Phosphoric acid	2.7.9.1	K01006	Pyruvate, orthophosphate dikinase	*ppdK*
C00009	TRINITY_DN2506_c0_g1	Phosphoric acid	2.5.1.72	K03517	Quinolinate synthase	*nadA*
C00009	TRINITY_DN4826_c0_g1	Phosphoric acid	3.1.3.2	K14410	Lysosomal acid phosphatase	*acp2*
C00009	TRINITY_DN37805_c0_g1	Phosphoric acid	3.1.3.2	K14379	Tartrate-resistant acid phosphatase type 5	*acp5*
C00163	TRINITY_DN2221_c0_g1	Propionic acid	6.2.1.1	K01895	Acetyl-CoA synthetase	*acss1_2*
C00163	TRINITY_DN1448_c0_g1	Propionic acid	6.2.1.1	K01895	Acetyl-CoA synthetase	*acss1_2*

C00163	TRINITY_DN1071_c1_g1	Propionic acid	6.2.1.1	K01895	Acetyl-CoA synthetase	*acss1_2*
C10462	TRINITY_DN3110_c0_g1	[6]-gingerol	2.1.1.-	K14857	AdoMet-dependent rRNA methyltransferase SPB1	*1spb1*
C10462	TRINITY_DN1378_c1_g4	[6]-gingerol	2.1.1.-	K00599	tRNA N(3)-methylcytidine methyltransferase METTL6	*mettl6*
C10462	TRINITY_DN2032_c0_g1	[6]-gingerol	2.1.1.-	K18162	NADH dehydrogenase [ubiquinone] 1 alpha subcomplex assembly factor 5	*ndufaf5*
C10462	TRINITY_DN476_c2_g1	[6]-gingerol	2.1.1.-	K14292	Trimethylguanosine synthase	*tgs1*
C10462	TRINITY_DN421_c0_g1	[6]-gingerol	2.1.1.-	K00599	tRNA N(3)-methylcytidine methyltransferase METTL6	*mettl6*
C10462	TRINITY_DN2583_c0_g1	[6]-gingerol	2.1.1.-	K17878	EEF1A N-terminal glycine/lysine methyltransferase	*nnt1*
C10462	TRINITY_DN4892_c0_g1	[6]-gingerol	2.1.1.-	K17878	EEF1A N-terminal glycine/lysine Methyltransferase	*nnt1*
C00311	TRINITY_DN8067_c0_g1	Isocitric acid	4.2.1.3	K01681	Aconitate hydratase	*aco*
C00311	TRINITY_DN2000_c0_g1	Isocitric acid	4.2.1.3	K01681	Aconitate hydratase	*aco*
C00311	TRINITY_DN951_c2_g1	Isocitric acid	4.1.3.1	K01637	Isocitrate lyase	*E4.1.3.1*
C00378	TRINITY_DN4826_c0_g1	Thiamine	3.1.3.2	K14410	Lysosomal acid phosphatase	*acp2*

C00378	TRINITY_DN37805_c0_g1	Thiamine	3.1.3.2	K14379	Tartrate-resistant acid phosphatase type 5	*Acp5*
C00147	TRINITY_DN3856_c0_g1	Adenine	2.4.2.7	K00759	Adenine phosphoribosyltransferase	*aprt*
C00530	TRINITY_DN3960_c0_g1	Hydroquinone	1.8.5.7	K07393	Glutathionyl-hydroquinone reductase	*ecm4*
C00530	TRINITY_DN2223_c0_g1	Hydroquinone	1.14.13.-	K06126	Ubiquinone biosynthesis monooxygenase Coq6	*coq6*
C00530	TRINITY_DN35784_c0_g1	Hydroquinone	3.1.1.-	K13806	Sn1-specific diacylglycerol lipase	*dagl*
C15603	TRINITY_DN1412_c0_g1	Hydroquinone	1.6.5.2	K03809	NAD(P)H dehydrogenase (quinone)	*wrbA*
C15603	TRINITY_DN1518_c1_g1	Hydroquinone	1.6.5.2	K03809	NAD(P)H dehydrogenase (quinone)	*wrbA*
C15603	TRINITY_DN83_c0_g1	Hydroquinone	1.3.5.2	K00254	Dihydroorotate dehydrogenase	*dhodh*
C15603	TRINITY_DN556_c1_g1	Hydroquinone	1.1.5.3	K00111	Glycerol-3-phosphate dehydrogenase	*glpA*
C15603	TRINITY_DN1494_c0_g1	Hydroquinone	1.3.5.1	K00235	Succinate dehydrogenase (ubiquinone) iron-sulfur subunit	*sdhb*
C15603	TRINITY_DN3177_c0_g1	Hydroquinone	1.3.5.1	K00235	Succinate dehydrogenase (ubiquinone) iron-sulfur subunit	*sdhb*
C15603	TRINITY_DN581_c0_g1	Hydroquinone	1.3.5.6	K00514	Zeta-carotene desaturase	*zds*
C00588	TRINITY_DN4327_c0_g1	Phosphocholine	3.1.4.3	K01114	Phospholipase C	*plc*
C00588	TRINITY_DN14661_c0_g2	Phosphocholine	3.1.4.3	K01114	Phospholipase C	*plc*
C00588	TRINITY_DN14661_c0_g1	Phosphocholine	3.1.4.3	K01114	Phospholipase C	*plc*

C00588	TRINITY_DN35322_c0_g1	Phosphocholine	2.7.7.15	K00968	Choline-phosphate cytidylyltransferase	*pcyt1*
C00588	TRINITY_DN3227_c0_g1	Phosphocholine	2.7.7.15	K00968	Choline-phosphate cytidylyltransferase	*pcyt1*
C00483	TRINITY_DN2223_c0_g1	Tyramine	1.14.13.-	K06126	Ubiquinone biosynthesis monooxygenase Coq6	*coq6*
C00483	TRINITY_DN38246_c0_g1	Tyramine	1.4.3.4	K00274	Monoamine oxidase	*mao*
C00116	TRINITY_DN689_c0_g1	Glycerol	2.7.1.30	K00864	Glycerol kinase	*glpK*
C00116	TRINITY_DN2467_c0_g1	Glycerol	3.1.1.23	K01054	Acylglycerol lipase	*mgll*
C16677	TRINITY_DN2223_c0_g1	All-trans-4-hydroxyretinoic acid	1.14.13.-	K06126	Ubiquinone biosynthesis monooxygenase Coq6	*coq6*
C00140	TRINITY_DN28793_c0_g1	N-acetyl-d-glucosamine	2.7.1.-	K00924	Kinase	*E2.7.1.-*
C00670	TRINITY_DN220_c0_g1	Glycerophosphocholine	3.1.4.46	K01126	Glycerophosphoryl diester phosphodiesterase	*glpQ*
C00670	TRINITY_DN43341_c0_g1	Glycerophosphocholine	3.1.1.5	K06130	Lysophospholipase II	*lpyla2*
C00559	TRINITY_DN2489_c0_g1	Deoxyadenosine	3.5.4.4	K01488	Adenosine deaminase	*add*
C00134	TRINITY_DN913_c0_g1	Putrescine	4.1.1.17	K01581	Ornithine decarboxylase	*odc1*
C00134	TRINITY_DN25200_c0_g1	Putrescine	4.1.1.17	K01581	Ornithine decarboxylase	*odc1*
C00134	TRINITY_DN5528_c0_g1	Putrescine	4.1.1.17	K01581	Ornithine decarboxylase	*odc1*
C00134	TRINITY_DN174_c0_g1	Putrescine	2.5.1.16	K00797	Spermidine synthase	*speE*

C00134	TRINITY_DN1400_c0_g1	Putrescine	2.5.1.16	K00797	Spermidine synthase	*speE*
C00134	TRINITY_DN3136_c0_g1	Putrescine	2.3.1.-	K14521	N-acetyltransferase 10	*nat10*
C00134	TRINITY_DN2569_c0_g1	Putrescine	2.3.1.-	K03372	Acetyl-CoA transportor	*slc33a1*
C00134	TRINITY_DN497_c5_g1	Putrescine	3.5.1.53	K12251	N-carbamoylputrescine amidase	*aguB*
C00134	TRINITY_DN7692_c0_g1	Putrescine	3.5.1.53	K12251	N-carbamoylputrescine amidase	*aguB*
C00170	TRINITY_DN2211_c0_g1	S-methyl-5'-thioadenosine	4.4.1.14	K01762	1-aminocyclopropane-1-carboxylate synthase	*acs*
C00170	TRINITY_DN678_c0_g1	S-methyl-5'-thioadenosine	4.4.1.14	K01762	1-aminocyclopropane-1-carboxylate synthase	*acs*
C00170	TRINITY_DN515_c0_g1	S-methyl-5'-thioadenosine	2.5.1.108	K07561	2-(3-amino-3-carboxypropyl)histidine synthase	*dph1*
C00170	TRINITY_DN174_c0_g1	S-methyl-5'-thioadenosine	2.5.1.16	K00797	Spermidine synthase	*speE*
C00170	TRINITY_DN1400_c0_g1	S-methyl-5'-thioadenosine	2.5.1.16	K00797	Spermidine synthase	*speE*
C00170	TRINITY_DN1849_c0_g1	S-methyl-5'-thioadenosine	2.5.1.79	K18787	Thermospermine synthase	*acl5*

C00170	TRINITY_DN1825_c0_g1	S-methyl-5'-thioadenosine	2.5.1.79	K18787	Thermospermine synthase	*acl5*
C00025	TRINITY_DN344_c6_g1	L-Glutamate	4.3.3.6	K06215	Pyridoxal 5'-phosphate synthase pdxS subunit	*pdxS*
C00025	TRINITY_DN2596_c0_g1	L-Glutamate	2.6.1.9	K00817	Histidinol-phosphate aminotransferase	*hisC*
C00025	TRINITY_DN3356_c0_g1	L-Glutamate	4.1.1.15	K01580	Glutamate decarboxylase	*gadB*
C00025	TRINITY_DN2634_c0_g1	L-Glutamate	6.3.4.2	K01937	CTP synthase	*pyrG*
C00025	TRINITY_DN7234_c0_g2	L-Glutamate	2.6.1.2	K00814	Alanine transaminase	*gpt*
C00025	TRINITY_DN560_c1_g1	L-Glutamate	2.6.1.2	K00814	Alanine transaminase	*gpt*
C00025	TRINITY_DN3286_c0_g1	L-Glutamate	2.6.1.2	K00814	Alanine transaminase	*gpt*
C00025	TRINITY_DN1114_c0_g1	L-Glutamate	2.3.1.1	K14682	Amino-acid N-acetyltransferase	*argAB*
C00025	TRINITY_DN4484_c0_g1	L-Glutamate	2.6.1.83	K10206	LL-diaminopimelate aminotransferase	*E2.6.1.83*
C00025	TRINITY_DN2207_c0_g1	L-Glutamate	6.1.1.17	K01885	Glutamyl-tRNA synthetase	*ears*
C00025	TRINITY_DN1100_c0_g7	L-Glutamate	6.1.1.17	K01885	Glutamyl-tRNA synthetase	*ears*
C00025	TRINITY_DN1100_c0_g1	L-Glutamate	6.1.1.17	K01885	Glutamyl-tRNA synthetase	*ears*
C00025	TRINITY_DN1303_c0_g1	L-Glutamate	6.3.5.3	K01952	Phosphoribosylformylglycinamidine synthase	*pfas*
C00025	TRINITY_DN1544_c0_g1	L-Glutamate	2.6.1.52	K00831	Phosphoserine aminotransferase	*serC*
C00025	TRINITY_DN1343_c0_g1	L-Glutamate	2.6.1.52	K00831	Phosphoserine aminotransferase	*serC*

C00025	TRINITY_DN40714_c0_g1	L-Glutamate	2.6.1.5	K00815	Tyrosine aminotransferase	*tat*
C00025	TRINITY_DN495_c0_g2	L-Glutamate	6.3.1.2	K01915	Glutamine synthetase	*glnA*
C00025	TRINITY_DN495_c0_g1	L-Glutamate	6.3.1.2	K01915	Glutamine synthetase	*glnA*
C00025	TRINITY_DN1550_c0_g1	L-Glutamate	6.3.1.2	K01915	Glutamine synthetase	*glnA*
C00025	TRINITY_DN111_c1_g1	L-Glutamate	6.3.1.2	K01915	Glutamine synthetase	*glnA*
C00025	TRINITY_DN3925_c0_g1	L-Glutamate	6.3.1.2	K01915	Glutamine synthetase	*glnA*
C00025	TRINITY_DN5897_c0_g1	L-Glutamate	2.6.1.42	K00826	Branched-chain amino acid aminotransferase	*ilvE*
C00025	TRINITY_DN552_c1_g1	L-Glutamate	2.6.1.42	K00826	Branched-chain amino acid aminotransferase	*ilvE*
C00025	TRINITY_DN934_c2_g1	L-Glutamate	2.6.1.42	K00826	Branched-chain amino acid aminotransferase	*ilvE*
C00025	TRINITY_DN1241_c0_g1	L-Glutamate	2.6.1.85	K13950	Para-aminobenzoate synthetase	*pabAB;*
C00025	TRINITY_DN4590_c0_g1	L-Glutamate	2.6.1.13	K00819	Ornithine--oxo-acid transaminase	*rocD*
C00025	TRINITY_DN671_c0_g1	L-Glutamate	2.6.1.13	K00819	Ornithine--oxo-acid transaminase	*rocD*
C00025	TRINITY_DN301_c2_g1	L-Glutamate	1.4.1.14	K00264	Glutamate synthase (NADH)	*glt1*
C00025	TRINITY_DN450_c4_g1	L-Glutamate	6.3.5.1	K01950	NAD+ synthase	*nadsyn1*
C00025	TRINITY_DN1895_c0_g1	L-Glutamate	6.3.2.2	K11204	Glutamate--cysteine ligase catalytic subunit	*gclc*
C00025	TRINITY_DN1655_c0_g1	L-Glutamate	2.6.1.16	K00820	Glutamine---fructose-6-phosphate transaminase	*glmS*
C00025	TRINITY_DN1430_c0_g1	L-Glutamate	6.3.5.4	K01953	Asparagine synthase	*asnB*

C00025	TRINITY_DN6105_c0_g2	L-Glutamate	1.4.1.3	K00261	Glutamate dehydrogenase	*glud1_2*
C00025	TRINITY_DN21539_c0_g1	L-Glutamate	1.4.1.3	K00261	Glutamate dehydrogenase	*glud1_2*
C00025	TRINITY_DN276_c0_g1	L-Glutamate	1.4.7.1	K00284	Glutamate synthase	*glu*
C00025	TRINITY_DN2374_c0_g1	L-Glutamate	2.6.1.1	K14455	Aspartate aminotransferase, mitochondrial	*got2*
C00025	TRINITY_DN298_c0_g1	L-Glutamate	2.6.1.1	K14455	Aspartate aminotransferase, mitochondrial	*got2*
C00025	TRINITY_DN4978_c0_g1	L-Glutamate	2.6.1.1	K00811	Aspartate aminotransferase, chloroplastic	*asp5*
C00025	TRINITY_DN2100_c0_g1	L-Glutamate	1.4.1.4	K00262	Glutamate dehydrogenase	*gdhA*
C00114	TRINITY_DN1168_c0_g1	Choline	3.1.1.7	K01049	Acetylcholinesterase	*ache*
C00114	TRINITY_DN303_c0_g1	Choline	2.6.-.-	K05290	GPI-anchor transamidase subunit K	*pigk*
C00114	TRINITY_DN220_c0_g1	Choline	3.1.4.46	K01126	Glycerophosphoryl diester phosphodiesterase	*glpQ*

Table S4. The gradient program of ultra-high performance liquid chromatography (UHPLC) used for sample separation.

Column	ACQUITY UPLC BEH Amide column (2.1 mm× 100 mm, 1.7 μm)		
Mobile phase	A: water + 25 mM Ammonium acetate + 25 mM Ammonium hydroxide<br>B: Acetonitrile		
Column Temperature	25°C		
Injection volume	2 μL		
Flow rate	0.3 mL min^{-1}		
Gradient	Duration (min)	A (%)	B (%)
	1.00	5.00	95.00
	13.00	35.00	65.00
	2.00	60.00	40.00
	0.10	5.00	95.00
	4.90	5.00	95.00
